# Representativeness of variation benchmark datasets

**DOI:** 10.1186/s12859-018-2478-6

**Published:** 2018-11-29

**Authors:** Gerard C. P. Schaafsma, Mauno Vihinen

**Affiliations:** Protein Structure and Bioinformatics, Department of Experimental Medical Science, Lund University, BMC B13, SE-221 84 Lund, Sweden

**Keywords:** Representativeness, Benchmark datasets, Variation, Variation interpretation, Mutation

## Abstract

**Background:**

Benchmark datasets are essential for both method development and performance assessment. These datasets have numerous requirements, representativeness being one. In the case of variant tolerance/pathogenicity prediction, representativeness means that the dataset covers the space of variations and their effects.

**Results:**

We performed the first analysis of the representativeness of variation benchmark datasets. We used statistical approaches to investigate how proteins in the benchmark datasets were representative for the entire human protein universe. We investigated the distributions of variants in chromosomes, protein structures, CATH domains and classes, Pfam protein families, Enzyme Commission (EC) classifications and Gene Ontology annotations in 24 datasets that have been used for training and testing variant tolerance prediction methods. All the datasets were available in VariBench or VariSNP databases. We tested also whether the pathogenic variant datasets contained neutral variants defined as those that have high minor allele frequency in the ExAC database. The distributions of variants over the chromosomes and proteins varied greatly between the datasets.

**Conclusions:**

None of the datasets was found to be well representative. Many of the tested datasets had quite good coverage of the different protein characteristics. Dataset size correlates to representativeness but only weakly to the performance of methods trained on them. The results imply that dataset representativeness is an important factor and should be taken into account in predictor development and testing.

**Electronic supplementary material:**

The online version of this article (10.1186/s12859-018-2478-6) contains supplementary material, which is available to authorized users.

## Background

Benchmark datasets are essential for method developers as well as for those who want to find the best performing tools. Benchmarks represent the golden standard of known cases. There are a number of requirements for benchmark datasets [1]. These include relevance, representativeness, non-redundancy, scalability, reusability, and cases must be experimentally verified and contain both positive and negative examples. The benchmark data should be relevant for the studied phenomenon to be able to capture its characteristics. Non-redundancy means in practice that overlapping cases are excluded to avoid bias. The data entries should be experimentally verified, not predicted. There must be both positive and negative examples. For applicability to systems of different sizes, the scalability is an important criterion. It is preferable to be able to reuse the dataset for different purposes. This is especially important since the collection and selection of high-quality datasets requires substantial amounts of work and effort.

The representativeness of a dataset means that the set covers the space of the investigated phenomenon i.e. provides a good and balanced cross-section of a population and includes the most information of the original dataset. What this means in practice depends on the area of the benchmark. In the case of variant tolerance/pathogenicity prediction, it means that the dataset represents the space of variations and their effects. Numerous tolerance prediction methods are based on supervised machine learning algorithms and are thus trained on known examples. The goal of these predictors is to learn to generalize from the given examples. If the examples used for training do not fully represent the phenomenon space, the performance of the tool will be negatively affected. Although representativeness is an important concept and relevant for many different types of studies and fields, it has not been fully defined. Similarity and likelihood were considered in the early attempts, subsequently, the theoretical background has been accounted e.g. based on the Bayesian [2] and fuzzy set approaches [3].

We have established the VariBench [1] and VariSNP [4] databases for variation benchmark datasets that have been used for training and testing of methods. Amino acid substitutions are among the most common disease-causing alterations due to genetic changes. Many methods have been developed for this domain [5] and are based on different principles. Evolutionary conservation measures are among the most useful features for such predictions. Some methods are based solely on sequence conservation and do not require machine learning approaches. These include e.g. SIFT [6], PROVEAN [7] and PANTHER [8]. Another group of methods utilizes machine learning (ML) algorithms. The features used for method training have just been considered to be useful or been selected with extensive feature selection approaches. Examples of this kind of tools are CADD [9], MutationAssessor [10], MutationTaster2 [11], PolyPhen-2 [12], PON-P2 [13] and VEST3 [14]. For certain tools, more than 1000 features representing amino acid physicochemical propensities, sequence conservation, variation position sequence context, protein structural features, Gene Ontology (GO) [15] annotations and others have been used.

The third category of predictors consists of meta-predictors, methods that use the predictions of other methods to make their own decisions. These tools are more advanced than a simple majority vote approach, which can be problematic [16]. Advanced ML-based meta-predictors include MetaLR [17], MetaSVM [17], and PON-P [18]. Methods in the fourth group, hybrid methods, combine diverse features and utilize evidence from experimental tests and e.g. clinical features. These tools are typically for specific applications of variants in a single or a few proteins e.g. for variants in BRCA1 and 2 sets [19–21], as well as in the mismatch repair system [22, 23].

Systematic method performance assessment requires in addition to benchmark test data also relevant measures. The required measures, their principles and applications have been discussed previously [24–26]. It is also important how the benchmark datasets are applied. A common problem has been circularity, i.e. the use of the same or similar data items for training and testing [27]. Several method assessments based on various benchmarks have been published [27–30].

We investigated the representativeness of datasets used for training and testing variant tolerance predictors that are available in VariBench and VariSNP. Since no similar studies have been reported, we had to start by determining which features capture the representativeness. We decided to investigate how well the available benchmark datasets represent the structural and functional characteristics on the human proteome. Vast differences were detected in the representativeness of the established variation datasets. We discuss the relevance of the representativeness for method performance and development.

## Methods and materials

### Datasets

In Table 1, an overview of the investigated benchmark datasets is provided.

**Table 1:** General properties of the investigated benchmark datasets

dataset	collection	subset of VariBench dataset	original filename	no. of variants	no. of variants mapped to PDB	% mapped to PDB	no. of variants in ExAC	% in ExAC
DS1	VariSNP		Neutral single nucleotide variants	446,013	39,081	8.76		
DS2	VariBench Dataset 1		Neutral_dbSNP_build_131_mapped	23,671	2358	9.96		
DS3	VariBenchDataset 1		Pathogenic_SNP_mapped	19,335	10,242	52.97	263	1.36
DS4	VariBench Dataset 2	1	Neutral_dataset_Olatubosun_et_al_with_mapping_annotated	19,459	2245	11.54		
DS5	VariBench Dataset 2	1	Pathogenic_training_dataset_from_PONP	14,610	7261	49.7	221	1.51
DS6	VariBench Dataset 4	1	Neutral_dataset_from_Thusberg_et_al_clustered_with_mapping	17,623	1743	9.89		
DS7	VariBench Dataset 4	1	Pathogenic_dataset_Thusberg_et_al_clustered_with_mapping	17,525	9519	54.32	227	1.30
DS8	VariBench Dataset 5	2	Neutral_dataset_Olatubosun_et_al_clustered_with_mapping	14,647	1706	11.65		
DS9	VariBench Dataset 5	2	Pathogenic_dataset_Olatubosun_et_al_clustered_with_mapping	13,096	6652	50.79	195	1.49
DS10	VariBench Dataset 7	2	Neutral_PON-P2_training_data	13,063	1731	13.25		
DS11	VariBench Dataset 7	2	Pathogenic_PON-P2_training_data	12,584	6420	51.02	173	1.37
DS12	VariBench Dataset 7	2	Neutral_PON-P2_test_data	1605	150	9.35		
DS13	VariBench Dataset 7	2	Pathogenic_PON-P2_test_data.csv	1301	481	36.97	23	1.77
DS14	VariBench Dataset 7	2	Neutral_PON-P2_c95_training	8664	953	11		
DS15	VariBench Dataset 7	2	Pathogenic_PON-P2_c95_training	7151	3728	52.13	81	1.13
DS16	VariBench Dataset 7	2	Neutral_PON-P2_c95_test	1053	82	7.79		
DS17	VariBench Dataset 7	2	Pathogenic_PON-P2_c95_test	751	272	36.22	12	1.60
DS18	VariBench Dataset 9		predictSNP_selected_tool_scores	16,098	4494	27.92		
DS19	VariBench Dataset 9		varibench_selected_tool_scores	10,266	3418	33.29		
DS20	VariBench Dataset 9		exovar_filtered_tool_scores	8850	2985	33.73		
DS21	VariBench Dataset 9		humvar_filtered_tool_scores	40,389	10,990	27.21		
DS22	PolyPhen-2		humvar-2011_12.neutral.humvar.output	21,151	2169	10.25		
DS23	PolyPhen-2		humvar-2011_12.deleterious.humvar.output	22,196	10,290	46.36	342	1.54
DS24	SwissVar		SwissVar_latest	75,042	12,749	16.99	16,049	21.39

Dataset 1 (DS1): neutral single amino acid substitutions (SAASs) from the VariSNP database [4]. The dataset contains 446,013 single nucleotide variants (SNVs) from dbSNP (build 149, GRCh38.p7) filtered to exclude disease-related variants found in ClinVar, Swiss-Prot or PhenCode (https://structure.bmc.lu.se/VariSNP/). The representativeness of the encoded protein variants was investigated.

Datasets 2-21 (DS2-DS21): protein tolerance predictor datasets. VariBench [1] contains information for experimentally verified effects and datasets that have been used for developing and testing the performance of prediction tools (https://structure.bmc.lu.se/VariBench/).

DS2: dataset comprising 23,671 human non-synonymous SNVs and associated SAASs for data from the dbSNP database build 131. Cases with insufficient data were removed from the original file.

DS3: pathogenic dataset of 19,335 SAASs obtained from the PhenCode database, IDbases and from 18 individual LSDBs.

DS2 and DS3 were used for the original predictor performance assessment [30].

DS4: subset of DS2 from which cancer cases were removed, 19,459 neutral non-synonymous coding SNVs and their SAASs.

DS5: subset of DS3 from which cancer cases were removed, 14,610 SAASs.

DS4 and DS5 were used for training PON-P [18].

DS6: subset of DS2 obtained by clustering the protein sequences based on their sequence similarity to remove close homologues which may cause problems with certain applications; 17,624 human non-synonymous coding SNVs and their SAASs on 6045 representative sequences (clusters).

DS7: subset of DS3 obtained as DS6; 17,525 SAASs on 954 representative protein sequences (clusters).

DS8: subset of DS4 obtained by clustering the protein sequences based on their sequence similarity to remove close homologues.

DS9: subset of DS5 obtained like DS8.

DS10: subset of DS4 filtered by the availability of features used in PON-P2.

DS11: subset of DS5, obtained like DS10.

DS12: subset of DS4 filtered by the availability of features used in PON-P2.

DS13: subset of DS5, obtained like DS12.

DS14-DS17: as DS10–13 with a probability of pathogenicity cutoff of 0.95.

DS10 and DS11 were used for training the PON-P2 predictor and DS12–13 for testing its performance [13].

DS18-DS21: Filtered versions of five publicly available benchmark datasets for pathogenicity prediction [27]. The sets contain variants from PredictSNP (DS18), VariBench (DS19), ExoVar (DS20), and HumVar (DS21).

DS22-DS23: PolyPhen-2 HumVar training datasets: 21,119 neutral (DS22) and 22,196 deleterious variations (DS23) in 9679 human proteins, no restriction for deleterious and neutral variations coming from the same proteins (ftp://genetics.bwh.harvard.edu/pph2/training). HumVar contains human variants associated with disease (except cancer variations) or loss of activity/function vs. common (minor allele frequency > 1%) human variation with no reported association with a disease or other effect [12].

DS24: 75,042 SwissVar variants (SAASs) downloaded (2017-04-19) from http://swissvar.expasy.org/cgi-bin/swissvar/result?format=tab, only those entries with a variant description were selected [31].

### Chromosomal distribution of variants

Python scripts (version 2.7.12) were developed to determine the number of variants per chromosome and total coding sequence (CDS) length in chromosomes. The observed numbers of variants per chromosome were taken from the datasets, expected numbers were weighted by the number of genes per chromosome or by length CDSs. The numbers of genes per chromosome were taken from the Ensembl Biomart service (http://www.ensembl.org/biomart/martview/) with the following settings for the number of genes: Ensembl Genes 89; Human genes (GRCh38.p10), Chromosome/scaffold 1–22, X, Y; Gene type: protein coding. Only unique results (for Gene Stable ID) were exported to a tab-delimited file. The total number of protein coding genes was 19,786. Settings for the CDS lengths were: Ensembl Genes 92; Human genes (GRCh38.p12), Chromosome/scaffold 1–22, X, Y; Attributes: Sequences. Peptide; Header information: Gene stable ID, Transcript stable ID, CDS length. Unique results only, were exported in FASTA format.

### Mapping to ExAC dataset

The cases in the pathogenic datasets were mapped to ExAC database (release 0.3.1) variants [32] with minor allele frequency (MAF) higher than 1%, but lower than 25%, in at least one of the seven geographical populations (Niroula and Vihinen, submitted). The dataset is available at http://structure.bmc.lu.se/VariBench/exac_aas.php.

### Mapping to PDB

To perform analyses related to CATH protein domains [33] and Pfam protein families [34], variants in the datasets were first mapped to PDB structures, using Python scripts. Depending on the level of the variant descriptions in the datasets (DNA or protein level) and/or the reference sequences (NCBI RefSeq, UniProtKB identifiers, Ensembl gene or protein identifiers), use was made of auxiliary files downloaded from the respective databases. Protein variant descriptions with a RefSeq reference sequence [35] or an Ensembl reference sequence [36] were first mapped to UniProt reference sequences [37]. A file containing cross-reference RefSeq-UniProt identifiers and UniProt sequence lengths was downloaded from UniProt (human and reviewed protein sequences, http://www.uniprot.org/uniprot/?query=*&fil=reviewed%3Ayes+AND+organism%3A%22Homo+sapiens+%28Human%29+%5B9606%5D%22). A file with cross-reference Ensembl-UniProt identifiers was obtained using the Ensembl Biomart service. Mapping was only done when the lengths of the RefSeq and the UniProt reference sequences matched.

Once variant descriptions were available on the protein level with a UniProt identifier for the reference sequence, residue level mapping to PDB structures was obtained from the pdb_chain_uniprot file, which was downloaded from the European Bioinformatics Institute (EBI) SIFTS FTP site (https://www.ebi.ac.uk/pdbe/docs/sifts/quick.html), including the start and end residues of the mapping using SEQRES, PDB sequence and UniProt numbering. When the protein was mapped to more than one PDB structure, the xml files were downloaded from the EBI FTP site (ftp.ebi.ac.uk/pub/databases/msd/sifts/split_xml/). If the residue on the position of the variant had the annotation ‘Not_Observed’, the structure was discarded. PDB structures were checked starting with those with the highest resolution. Resolution data were downloaded from the EBI site (http://www.ebi.ac.uk/pdbe/entry/search/index?organism_synonyms:“Homo sapiens (Human)”). When variants mapped to more than one chain in the same PDB structure, the first one was taken, and no further checking was done.

### CATH domains

For allocating and mapping variant positions to CATH domains, two files were downloaded from the CATH website (http://www.cathdb.info/download): CathDomainList.v4.1.0 containing all classified protein domains in CATH for class 1 (mainly alpha), class 2 (mainly beta), class 3 (alpha and beta) and class 4 (few secondary structures), and CathDomall.v4.1.0 in which domain boundaries for entries in the CATH database were described. Only variants which had been mapped to a PDB structure were used in the analysis.

To compare the CATH superfamily distributions in the datasets to the CATH superfamily space, a representative set of protein chains was obtained from the Research Collaboratory for Structural Bioinformatics (RCSB) PDB (ftp://resources.rcsb.org/sequence/clusters/). A file with sequence clusters with 95% identity (bc-95.out) was used to reduce redundancy by leaving only one representative for proteins with (almost) identical sequences. From each cluster (12,583 in total), the first sequence (chain) was taken as a representative, and the frequencies of CATH superfamilies were determined for each domain in that chain. There are 907 CATH superfamilies, to which 9572 CATH domains found in the 12,583 representative protein sequences could be allocated. These data were used as the background distribution for the analysis of the datasets.

### Pfam protein families

For mapping variant positions to Pfam families, a file was downloaded from UniProt with UniProt-Pfam cross references for human protein sequences. Equivalent to the PDB mapping, variant descriptions with a RefSeq or Ensembl reference sequence were first mapped to a UniProt protein sequence. Then Pfam IDs were looked up in the UniProt-Pfam cross references file, and for each Pfam domain the coordinates were obtained from the corresponding UniProtID.xml file. These corresponding xml files were downloaded from the Pfam database at http://pfam.xfam.org/. If the position of the variant was within the coordinates of a Pfam domain, it was counted.

To compare the Pfam domain distribution in the datasets, the frequencies of Pfam domains in the UniProt-Pfam cross references download were determined.

### Enzyme commission numbers

Cross references for UniProt ID and Enzyme Commission (EC) numbers [38] for all human proteins were downloaded from UniProt and served as reference dataset. The frequencies of the EC numbers in the reference set and the datasets were determined on all 4 levels.

Equivalent to the PDB mapping, variant descriptions with a RefSeq or Ensembl reference sequence were first mapped to a UniProt protein sequence. Then using the UniProt-EC numbers cross-references, EC numbers were allocated to each variant in the datasets, when applicable.

### Gene ontology terms

Cross references for UniProt ID and GO terms, including the identifiers for the 3 domains/aspects of the GO (MF: Molecular Function, BP: Biological Process and CC: Cellular Component), were obtained using the QuickGO service at the EBI website (http://www.ebi.ac.uk/QuickGO/GAnnotation) using the UniProt identifiers from the cross-reference RefSeq-UniProt file.

Variant descriptions with a RefSeq or Ensembl reference sequence were first mapped to a UniProt protein sequence. Then using the UniProt-GO identifiers cross-references, GO terms were allocated to each variant in the datasets, where applicable.

### Statistical tests

Pearson’s chi-square test (SciPy package v.0.19.0, scipy.stats.chisquare) was used to compare the distribution of variants over all chromosomes in the datasets (the observed numbers) to the expected numbers. For the statistical test of the chromosomal distribution, a two-tailed binomial test (SciPy package v.0.19.0, scipy.stats.binom_test) was used. The distributions of CATH, Pfam, EC and GO classes at each level were tested using the Python implementation (SciPy package v.0.19.0, scipy.stats.ks_2samp) of the Kolmogorov-Smirnov (KS) 2-sample test.

To estimate how well the datasets represented the classes within the classification schemes the coverage was calculated as follows

$$ coverage=\frac{A(DS)}{A}, $$

where *A(DS)* is the number of class labels in the dataset *DS* and *A* is the total number of classes in the classification system. A class is covered if and only if at least one representative belongs to the class.

## Results

To test the representativeness of the variant datasets statistical analyses were performed to reveal how well the datasets covered the overall distribution in the human proteome.

### Inclusion of benign variants to datasets for pathogenic variants

First, we investigated the relevance of the datasets. This was done for benign variants obtained from the ExAC database, which contains information for allele frequencies of 63,197 variants from 60,706 individuals. We included only variants that have 1% < MAF < 25% in at least one population, as frequent variants are considered to be benign. This is a reasonable and widely used assumption, however, a small number of highly frequent variants are known to be disease-associated e.g. in late onset conditions or in mild diseases. The cases in the pathogenic variant datasets were mapped to the ExAC entries.

Datasets of pathogenic variants contained only 1.13 to 1.77% of benign variants (Table 1) except for the SwissVar dataset that contains both benign and pathogenic variants. The percentage of benign cases is 21.39% in this dataset. The selection of pathogenic variants from SwissVar contains neutral variants at about the same frequency as the other datasets (1.31). The ratio of benign variants in the pathogenic datasets is so small that it does not bias methods developed based on them. It has to be remembered that phenotype is not a binary trait, instead has a continuum as described in the pathogenicity model [39]. In conclusion, the pathogenic datasets contain only minor amount of benign cases and thus be considered to contain relevant cases.

### Mapping to PDB

The results for mapping of variants to a PDB structure are given in Table 1. Variant mapping rates to PDB structures ranges from 7.8% for the cases in DS1 to 54% for DS7 (Table 1). The ratio of mapped variants in the pathogenic datasets was always higher (36–54%) than for the neutral counterparts (8–13%). These differences are partially correlated with the mapping to a UniProt protein sequence, which shows the same pattern. This is to be expected, since to be able to map to a PDB structure a UniProt ID is needed, on the other hand, not every UniProt ID is mapped to PDB structure(s). Every variant position cannot be mapped to a PDB sequence, since the coverage of UniProt sequences in PDB structures can differ greatly (1–100%). The termini of the proteins are often too flexible to be seen in the structures and cannot therefore be mapped to structures. There can also be gaps in the structures, especially in loop regions. Many structures are for part of the entire protein covering one or more domains.

The large difference between DS2 (neutral variants, 10% mapped) and DS3 (pathogenic variants, 53% mapped) seems to be negatively associated with the large difference in the number of protein sequences the variants could be mapped to (DS2: 7230; DS3: 1182; Table 2). Disease-related variants have a non-random distribution. Further, they have been extensively investigated in certain genes/proteins and diseases. For instance, the maximum number of variants mapped to a UniProt sequence is 2294 in DS3 (P04637; cellular tumor antigen p53), whereas this number in DS2 is only 71 (P20929; nebulin). The way the datasets were constructed can also play a role: DS2 is a selection of human non-synonymous coding SNVs from dbSNP (allele frequency > 0.01 and chromosome sample count > 49, and filtered for disease-associated SNVs), whereas its pathogenic counterpart, DS3, was selected from the PhenCode database [40], IDbases [41], and 18 additional LSDBs, all of which contained a substantial number of variants.

**Table 2:** Mapping of the datasets to UniProt protein sequences

dataset	no. of unique UniProt protein sequences	no. of variants mapped to a UniProt sequence	% variants mapped	maximum no. of variants mapped to a UniProt sequence	UniProt ID with maximum no. of variants	protein name	gene
DS1	17,571	378,706	84.9	1451	Q8WZ42	Titin	*TTN*
DS2	7230	18,660	78.8	71	P20929	Nebulin	*NEB*
DS3	1182	19,318	99.9	2294	P04637	Cellular tumor antigen p53	*TP53*
DS4	6541	15,880	81.6	56	P46013	Proliferation marker protein Ki-67	*MKI67*
DS5	1093	14,597	99.9	382	P00451	Coagulation factor VIII	*F8*
DS6	4895	13,811	78.4	71	P20929	Nebulin	*NEB*
DS7	953	17,514	99.9	2294	P04637	Cellular tumor antigen p53	*TP53*
DS8	4517	11,847	80.9	56	P46013	Proliferation marker protein Ki-67	*MKI67*
DS9	884	13,096	100.0	382	P00451	Coagulation factor VIII	*F8*
DS10	4997	10,882	83.3	27	Q86WI1	Fybrocystin-L	*PKHD1L1*
DS11	979	12,584	100.0	378	P00451	Coagulation factor VIII	*F8*
DS12	545	1288	80.2	14	Q13576	Ras GTPase-activating-like protein IQGAP2	*IQGAP2*
DS13	90	1301	100.0	100	P04839	Cytochrome b-245 heavy chain	*CYBB*
DS14	3799	7185	82.9	26	Q86WI1	Fybrocystin-L	*PKHD1L1*
DS15	785	7151	100.0	196	P00439	Phenylalanine-4-hydroxylase	*PAH*
DS16	424	848	80.5	11	Q8NEM0	Microcephalin	*MCPH1*
DS17	72	751	100.0	89	P04839	Cytochrome b-245 heavy chain	*CYBB*
DS18	3278	12,056	74.9	363	P00451	Coagulation factor VIII	*F8*
DS19	4129	10,154	98.9	1799	P04637	Cellular tumor antigen p53	*TP53*
DS20	3509	8662	97.9	137	P68871	Hemoglobin subunit beta	*HBB*
DS21	9038	39,735	98.4	460	P00451	Coagulation factor VIII	*F8*
DS22	8791	21,151	100.0	48	P20930, Q7Z442	Filaggrin, Polycystic kidney disease protein 1-like 2	*FLG*, *PKD1L2*
DS23	1852	22,196	100.0	472	P00451	Coagulation factor VIII	*F8*
DS24	12,735	75,042	100.0	1338	P04637	Cellular tumor antigen p53	*TP53*

DS4-DS17 are subsets of DS2 and DS3, thus similarities to the parent databases are expected. DS22 (neutral) and DS23 (pathogenic) also show similar patterns: high number of unique UniProt sequences, low maximum number of variants mapped to a specific protein in the neutral set; and the opposite situation for the pathogenic set.

DS18-DS21, mixed datasets of both neutral and pathogenic variants, all show mapping of approximately 30% of variants (Table 1). This is close to the means of the neutral and pathogenic datasets. For instance, the mean of the percentages mapped to PDB for DS22 and DS23 is 28%, and for DS21, which is a selection of the combination of DS22 and DS23, it is 27%. DS24, also a mixed dataset, had a rather low percentage, 17%. When comparing the mapping percentages of these datasets to the ratios pathogenic to total, which were in the range of 0.42 for DS19 to 0.62 for DS18 [27] and 0.44 for DS24 (data from 2017 to 07-06), we did not find a clear correlation.

### Chromosomal distribution of variants in the datasets

The chromosomal distribution of variants based on numbers of genes in chromosomes in DS1 is shown in Table 3, the results for the other datasets are in Tables S1-S23 (Additional file 1). The summary of results in Table 4 shows that DS16 has the highest number of chromosomes, 13, with an unbiased distribution of variants, whereas DS24 showed the lowest number, 2. The neutral VariBench datasets (DS1, DS2, DS4, DS6, DS8, DS10, DS12, DS14, DS16, DS22) always had higher numbers of chromosomes (range 7–13, mean 9.3) with an unbiased distribution than their pathogenic counterparts (DS3, DS5, DS7, DS9, DS11, DS13, DS15, DS17, DS23), range 3–6 chromosomes, mean 4.3. Since DS14-DS17 are subsets of DS2 and DS3, seeing the same difference between the neutral and pathogenic datasets is not surprising, although it would depend on how the subsets are selected. The comparison of datasets with their subsets seems to support this. For DS2 and DS3, differences with their subsets DS4-DS17 were in most cases no more than one chromosome, except for DS3 where the number of chromosomes with an unbiased variant distribution in the subset is sometimes even double (DS3 compared to subsets DS9 and DS17) (Table 4).

**Table 3:** Analysis of the chromosomal distribution of variants in dataset DS1

Chromosome	no. of genes	CDS length	no. of observed variants	no. of expected variants (no. of genes)	no. of expected variants (CDS length)	p-value^a^ (no of genes)	p-value^a^ (CDS length)
1	2037	3,483,903	45,856	45,915	45,339	0.773155	0.010565
2	1238	2,517,642	31,391	27,905	32,765	< 10^−4^	< 10–4
3	1071	1,965,098	24,735	24,141	25,574	< 10^−4^	< 10–4
4	745	1,365,661	16,936	16,793	17,773	0.260634	< 10–4
5	882	1,601,648	19,148	19,881	20,844	< 10^−4^	< 10–4
6	1035	1,735,760	22,495	23,330	22,589	< 10^− 4^	0.523159
7	901	1,609,177	21,764	20,309	20,942	< 10^−4^	< 10–4
8	668	1,135,640	16,239	15,057	14,779	< 10^−4^	< 10–4
9	770	1,382,150	19,117	17,356	17,987	< 10^− 4^	< 10–4
10	727	1,322,286	17,489	16,387	17,208	< 10^−4^	0.0292
11	1278	2,005,315	28,704	28,807	26,097	0.532354	< 10–4
12	1033	1,776,908	20,797	23,284	23,125	< 10^−4^	< 10–4
13	324	634,435	7401	7303	8257	0.247573	< 10–4
14	614	1,079,560	13,972	13,840	14,049	0.254342	0.511939
15	589	1,189,858	14,846	13,276	15,485	< 10^−4^	< 10–4
16	858	1,451,775	22,351	19,340	18,893	< 10^−4^	< 10–4
17	1184	1,971,211	26,518	26,688	25,653	0.284589	< 10–4
18	268	534,152	6644	6041	6951	< 10^−4^	0.000187
19	1467	2,277,812	34,032	33,067	29,643	< 10^−4^	< 10–4
20	540	811,690	11,340	12,172	10,563	< 10^−4^	< 10–4
21	233	342,226	5194	5252	4454	0.424789	< 10–4
22	439	712,404	10,412	9895	9271	< 10^−4^	< 10–4
X	840	1,296,174	8557	18,934	16,868	< 10^−4^	< 10–4
Y	45	67,500	51	1014	878	< 10^−4^	0.010565

^a^results of binomial test

**Table 4:** Summary of the chromosomal distributions in the datasets. Chromosomes with non-biased distribution are indicated by an asterisk

	chromosome																								
dataset	1	2	3	4	5	6	7	8	9	10	11	12	13	14	15	16	17	18	19	20	21	22	X	Y	no. of chromosomes
DS1	*			*							*		*	*			*				*				7
DS2	*							*	*		*	*	*		*					*					8
DS3												*		*		*									3
DS4	*							*	*		*	*	*		*	*				*					9
DS5							*						*		*		*							*	5
DS6	*							*	*			*	*		*	*				*		*			9
DS7												*		*		*									3
DS8	*				*				*			*			*	*						*			7
DS9							*						*	*	*		*							*	6
DS10						*	*	*	*		*	*			*	*						*			9
DS11										*					*	*	*							*	5
DS12	*			*			*		*				*					*		*	*	*			9
DS13		*													*									*	3
DS14						*	*	*			*	*	*	*	*	*				*		*			11
DS15														*	*	*		*						*	5
DS16	*			*			*		*	*		*	*	*	*			*			*	*		*	13
DS17		*									*		*		*			*						*	6
DS18				*							*	*	*			*		*							6
DS19			*							*	*			*	*										5
DS20	*	*	*			*		*				*	*	*	*	*						*			11
DS21	*		*												*	*									4
DS22		*	*	*	*			*	*					*	*				*	*	*				11
DS23			*												*		*								3
DS24															*						*				2
no. of datasets	9	4	5	5	2	3	6	7	8	3	8	11	12	10	19	12	5	5	1	6	5	7	0	7	

For the PON-P2 training and test datasets, DS10-DS13, their subsets DS14-DS17 were all generated with the same selection criterion, 95% probability of pathogenicity cutoff. In all but one (3 out of 4) case the subset has a higher number of chromosomes with an unbiased distribution. DS10 compared to its subset DS14, number of chromosomes with unbiased distribution are 9 and 11, respectively. DS11 compared to DS15, number of chromosomes with unbiased distribution is 5 for both. DS12 compared to DS16, number of chromosomes with unbiased distribution are 9 and 13, respectively. DS13 compared to DS17, number of chromosomes with unbiased distribution is 3 and 6, respectively. Most subsets, except DS15, had a higher number of chromosomes with an unbiased distribution than their parent datasets (Table 4).

The number of chromosomes with an unbiased distribution for DS21, which is a subset of the combined DS22 and DS23, is 4, comparable to the 3 chromosomes for the pathogenic DS23 (Table 4). The numbers for the mixed datasets (DS18, DS19, DS20, DS21 and DS24) were like those for the pathogenic datasets, range 2–6 chromosomes, mean 4.2. The numbers for the X chromosome are strongly biased for the pathogenic datasets. Mendelian diseases with defects in this chromosome have complete penetrance. One would expect to see the same for the Y chromosome, but that is not the case. The results for the Y chromosome are based on very low numbers compared to the other chromosomes. The numbers for chromosome 19 are also very biased, apart from DS22.

The distribution of variants in the whole human genome over the 24 chromosomes was also tested. Pearson’s chi square test statistic for the number of variants over all 24 chromosomes in DS1 was 8657.11 (*p* < 10^− 4^), so the distribution of the variants over all chromosomes is biased. The results for the other datasets are in Table S24 (Additional file 1).

Chromosomal distribution was studied also by comparing to the coding region length in chromosomes (Table 3 and Additional file 1: Tables S1-S23). The results are not identical but show similar trends as gene number based statistics. The differences between the two studies are most apparent in some of the smallest datasets, where one or a few exceptionally long or short genes can have a big effect on the total CDS length.

### Domain and superfamily distribution of variants

**Fig. 1:**
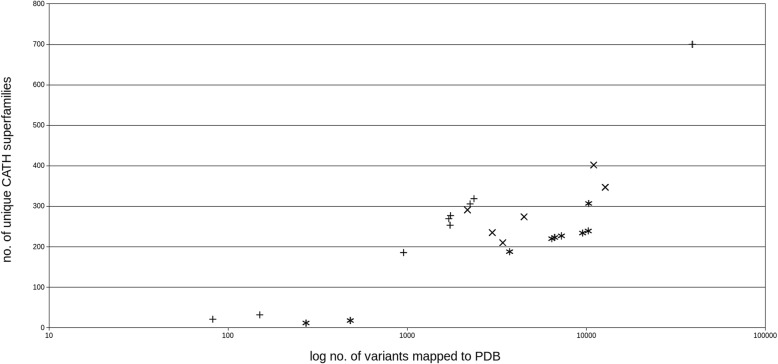
Number of unique CATH domains in relation to the log number of variants mapped to a PDB structure in each dataset. +: neutral datasets, *: pathogenic datasets, x: mixed datasets. The largest dataset, DS1, had also the largest number of unique CATH superfamilies, and there seems to be a positive correlation between the number of mapped variants and the number of unique CATH superfamilies.

The numbers of variants mapped to CATH domains, the numbers of variants with a CATH classification (superfamily) and the numbers of unique CATH superfamilies found in each dataset are provided in Table 5. The number of unique CATH superfamilies is plotted against the log number of variants mapped to a PDB structure in Fig. 1.

**Table 5:** Mapping of the datasets to PDB structures and CATH domains

dataset	no. of variants mapped to PDB	no. of variants mapped to CATH domain	% mapped to CATH domain (of mapped to PDB)	no. of variants with a CATH classification	% with a CATH classification (of mapped to PDB)	no. of unique CATH superfamilies
DS1	39,081	23,303	59.63	21,853	55.92	700
DS2	2358	1387	58.82	1319	55.94	319
DS3	10,242	6580	64.25	6396	62.45	239
DS4	2245	1325	59.02	1262	56.21	306
DS5	7261	4687	64.55	4556	62.75	227
DS6	1743	991	56.86	941	53.99	277
DS7	9519	6100	64.08	5920	62.19	234
DS8	1706	973	57.03	928	54.40	269
DS9	6652	4301	64.66	4170	62.69	223
DS10	1731	865	49.97	826	47.72	253
DS11	6420	4350	67.76	4212	65.61	220
DS12	150	66	44.00	62	41.33	32
DS13	481	142	29.52	135	28.07	18
DS14	953	478	50.16	454	47.64	186
DS15	3728	2557	68.59	2486	66.68	188
DS16	82	38	46.34	36	43.90	21
DS17	272	78	28.68	73	26.84	12
DS18	4494	2980	66.31	2862	63.68	274
DS19	3418	2081	60.88	2035	59.54	210
DS20	2985	2086	69.88	2031	68.04	235
DS21	10,990	7051	64.16	6786	61.75	402
DS22	2169	1301	59.98	1217	56.11	291
DS23	10,290	6566	63.81	6353	61.74	307
DS24	12,749	7828	61.40	7499	58.82	347

The percentages of variants mapped to CATH domains ranged from 29.5% for DS13 to 69.9% for DS20, the percentages of variants with a CATH classification ranged from 26.8% (DS13) to 68.1% (DS20), Table 5. The percentages for the pathogenic datasets are in general higher than those for their neutral counterparts, both for the mapping to CATH domains as well as for the CATH classifications. Exceptions are DS12 and DS13 and their subsets DS16 and DS17, where the situation is the opposite. These datasets contain low numbers of variants with CATH classifications. The mixed datasets (DS18-DS21 and DS24) have percentages (mean 64.52% for CATH domains) close to the mean percentage of the pathogenic datasets without the values for DS13 and DS17 (mean 65.39%). This is similar for the CATH classification: mean is 65.38% for the pathogenic datasets without DS13 and DS17, mean for the mixed datasets is 62.36%.

CATH classifies structures on four levels Class, Architecture, Topology and Homology. We investigated the distribution of variants to these categories by using the KS test (Table 6). On the Class level, the null hypothesis was not rejected for any dataset (*p* > 0.05). On the Architecture level, only the DS12, DS13, DS16 and DS17 showed biased distributions. DS16 and DS17 are subsets of DS12 and DS13, respectively. DS13 and DS17 are the smallest investigated ones with 1301 and 751 variants, respectively. On the Topology and Homology levels, all datasets have biased distributions.

**Table 6:** Kolmogorov-Smirnov 2-sample test statistics (KS) for each dataset on the Class, Architecture, Topology and Homology levels of CATH superfamilies

dataset	KS Class	KS Architecture	KS Topology	KS Homology
DS1	0.25 (0.99688)^a^	0.17 (0.76005)	0.30 (< 10^−4^)	0.36 (< 10^−4^)
DS2	0.25 (0.99688)	0.20 (0.53720)	0.60 (< 10^−4^)	0.65 (< 10^−4^)
DS3	0.25 (0.99688)	0.33 (0.05499)	0.68 (< 10^−4^)	0.74 (< 10^−4^)
DS4	0.25 (0.99688)	0.23 (0.34203)	0.61 (< 10^− 4^)	0.66 (< 10^− 4^)
DS5	0.25 (0.99688)	0.30 (0.10884)	0.69 (< 10^−4^)	0.75 (< 10^−4^)
DS6	0.25 (0.99688)	0.17 (0.76005)	0.65 (< 10^− 4^)	0.70 (< 10^− 4^)
DS7	0.25 (0.99688)	0.33 (0.05499)	0.68 (< 10^−4^)	0.74 (< 10^− 4^)
DS8	0.25 (0.99688)	0.20 (0.53720)	0.65 (< 10^−4^)	0.70 (< 10^− 4^)
DS9	0.25 (0.99688)	0.30 (0.10884)	0.69 (< 10^− 4^)	0.75 (< 10^− 4^)
DS10	0.25 (0.99688)	0.20 (0.53720)	0.67 (< 10^− 4^)	0.72 (< 10^− 4^)
DS11	0.25 (0.99688)	0.30 (0.10884)	0.70 (< 10^− 4^)	0.76 (< 10^− 4^)
DS12	0.25 (0.99688)	0.50 (0.00062)	0.94 (< 10^− 4^)	0.96 (< 10^− 4^)
DS13	0.50 (0.53344)	0.73 (< 10^− 4^)	0.97 (< 10^− 4^)	0.98 (< 10^− 4^)
DS14	0.25 (0.99688)	0.23 (0.34203)	0.75 (< 10^− 4^)	0.79 (< 10^− 4^)
DS15	0.25 (0.99688)	0.33 (0.05499)	0.73 (< 10^− 4^)	0.79 (< 10^− 4^)
DS16	0.25 (0.99688)	0.67 (< 10^− 4^)	0.96 (< 10^− 4^)	0.98 (< 10^− 4^)
DS17	0.50 (0.53344)	0.80 (< 10^− 4^)	0.98 (< 10^− 4^)	0.99 (< 10^− 4^)
DS18	0.25 (0.99688)	0.17 (0.76005)	0.64 (< 10^− 4^)	0.70 (< 10^− 4^)
DS19	0.25 (0.99688)	0.27 (0.20033)	0.72 (< 10^− 4^)	0.77 (< 10^− 4^)
DS20	0.25 (0.99688)	0.17 (0.76005)	0.68 (< 10^− 4^)	0.74 (< 10^− 4^)
DS21	0.25 (0.99688)	0.17 (0.76005)	0.49 (< 10^− 4^)	0.56 (< 10^− 4^)
DS22	0.25 (0.99688)	0.20 (0.53720)	0.61 (< 10^− 4^)	0.68 (< 10^− 4^)
DS23	0.25 (0.99688)	0.27 (0.2003)	0.60 (< 10^− 4^)	0.66 (< 10^− 4^)
DS24	0.25 (0.99688)	0.23 (0.34203)	0.56 (< 10^− 4^)	0.62 (< 10^− 4^)

^a^*p*-value in brackets

For the human proteome, there are 4 classes, 30 architectures, 508 topologies and 907 superfamilies in CATH. The maximum numbers for the datasets are 4, 30, 419, and 700, respectively. The numbers of mapped CATH superfamilies are generally in the order of 200 to 400, the minimum being 12 and the maximum 700. Although far from complete, the spread to the CATH levels indicates inclusion of numerous types of proteins among the datasets. The coverages on the levels 3 and 4 range from 1.8 to 82.5% and from 1.3 to 77.2%, respectively (Table 7).

**Table 7:** Coverage of proteins and all features compared to reference (%)

dataset	UniProt	CATH 1st level	CATH 2nd level	CATH 3rd level	CATH 4th level	Pfam	EC 1st level	EC 2nd level	EC 3rd level	EC 4th level	GO
DS1	86.98	100.00	100.00	82.48	77.18	90.77	100.00	98.18	99.41	99.15	98.33
DS2	35.79	100.00	83.33	40.35	35.17	36.01	100.00	90.91	85.29	61.61	72.97
DS3	5.85	100.00	70.00	32.48	26.35	13.85	100.00	76.36	61.18	24.92	46.03
DS4	32.38	100.00	83.33	39.17	33.74	34.16	100.00	89.09	84.71	60.14	70.93
DS5	5.41	100.00	70.00	30.91	25.03	12.94	100.00	76.36	60.59	23.99	44.55
DS6	24.23	100.00	83.33	35.24	30.54	33.10	100.00	89.09	83.53	49.23	63.13
DS7	4.72	100.00	70.00	32.09	25.80	13.52	100.00	76.36	60.59	22.99	42.61
DS8	22.36	100.00	83.33	34.84	29.66	31.64	100.00	87.27	82.94	48.45	61.76
DS9	4.38	100.00	70.00	30.51	24.59	12.68	100.00	76.36	60.00	22.21	41.55
DS10	24.74	100.00	80.00	32.87	27.89	28.46	100.00	87.27	81.76	51.24	62.60
DS11	4.85	100.00	70.00	30.31	24.26	11.65	100.00	72.73	58.24	23.22	42.15
DS12	2.70	75.00	50.00	5.51	3.53	2.56	83.33	27.27	14.71	2.79	18.11
DS13	0.45	75.00	26.67	2.95	1.98	1.40	66.67	18.18	8.24	1.01	9.00
DS14	18.81	100.00	76.67	25.20	20.51	20.93	100.00	78.18	70.00	37.38	51.87
DS15	3.89	100.00	66.67	27.17	20.73	9.61	100.00	70.91	55.88	20.43	38.21
DS16	2.10	75.00	33.33	3.54	2.32	2.02	66.67	25.45	12.94	2.48	14.72
DS17	0.36	75.00	20.00	1.77	1.32	1.12	66.67	14.55	7.06	0.77	8.27
DS18	16.23	100.00	83.33	36.02	30.21	22.29	100.00	85.45	80.00	38.24	58.66
DS19	20.44	100.00	76.67	28.15	23.15	21.99	100.00	85.45	74.12	35.22	58.93
DS20	17.37	100.00	83.33	31.89	25.91	19.24	100.00	89.09	75.88	38.78	59.35
DS21	44.74	100.00	90.00	50.98	44.32	42.19	100.00	94.55	90.59	71.13	82.17
DS22	43.52	100.00	90.00	39.17	32.08	36.55	100.00	90.91	86.47	61.53	75.51
DS23	9.17	100.00	80.00	39.96	33.85	18.77	100.00	87.27	75.29	35.76	55.21
DS24	63.04	100.00	90.00	44.49	38.26	58.14	100.00	96.36	94.71	85.53	91.74

### Protein family distribution of variant datasets

Pfam [34] is a widely-used classification for protein domains. The numbers and distribution of Pfam domains per dataset are depicted in Table 8. Pfam classifies protein families based on sequence similarities. The reference data for 5734 Pfam domains and their frequencies in the entire human proteome are in Additional file 2. Out of the 20,201 reviewed proteins in UniProt representing the human proteome in our study, 17,340 sequences (86%) had cross references to one or more Pfam domains.

**Table 8:** Mapping of the datasets to Pfam domains

dataset	number of unique Pfam domains	number of variants with a Pfam domain	% variants with a Pfam domain of total number of variants in dataset	no. of variants mapped to a UniProt sequence	% variants with a Pfam domain of number of variants mapped to UniProt	KS statistic^a^
DS1	5213	148,681	33.34	378,706	39.26	0.25 (< 10^− 4^)
DS2	2065	7307	30.87	18,660	39.16	0.64 (< 10^− 4^)
DS3	794	14,228	73.59	19,318	73.65	0.86 (< 10^− 4^)
DS4	1954	6589	33.86	15,880	41.49	0.66 (< 10^− 4^)
DS5	742	10,997	75.27	14,597	75.34	0.87 (< 10^− 4^)
DS6	1898	5293	30.03	13,811	38.32	0.67 (< 10^− 4^)
DS7	775	12,842	73.28	17,514	73.32	0.86 (< 10^− 4^)
DS8	1810	4833	33.00	11,847	40.80	0.68 (< 10^−4^)
DS9	727	9796	74.80	13,096	74.80	0.87 (< 10^− 4^)
DS10	1632	4396	33.65	10,882	40.40	0.72 (< 10^−4^)
DS11	668	9641	76.61	12,584	76.61	0.88 (< 10^− 4^)
DS12	147	579	36.07	1288	44.95	0.97 (< 10^−4^)
DS13	80	897	68.95	1301	68.95	0.99 (< 10^− 4^)
DS14	1197	2656	30.66	7185	36.97	0.79 (< 10^− 4^)
DS15	551	5619	78.85	7151	80.31	0.90 (< 10^− 4^)
DS16	116	354	33.62	848	42.22	0.98 (< 10^−4^)
DS17	64	526	70.04	751	70.04	0.99 (< 10^−4^)
DS18	1265	7190	44.66	12,056	59.64	0.78 (< 10^−4^)
DS19	1172	4859	47.33	10,154	47.85	0.80 (< 10^−4^)
DS20	1046	4818	54.44	8662	55.62	0.82 (< 10^− 4^)
DS21	2301	20,415	50.55	39,735	51.38	0.60 (< 10^− 4^)
DS22	2090	7727	36.53	21,151	36.53	0.64 (< 10^−4^)
DS23	1073	16,309	73.48	22,196	73.48	0.81 (< 10^− 4^)
DS24	3325	41,997	55.94	75,042	55.96	0.61 (< 10^−4^)

^a^*p*-value between brackets

The proportion of variants to which a Pfam domain could be allocated is dependent on the fraction of variants mapped to a UniProt sequence, ranging from 75% (Table 2, DS18) to 100% (Table 2, DS9, DS11, DS13, DS15, DS17, DS22, DS23, DS24). DS22 contains the lowest fraction of variants within Pfam domains (Tables 8, 36.8%), whereas DS15 showed the highest number (80.3%). The percentages for the neutral datasets were always lower (mean 40.6%) than those for the pathogenic datasets, mean 74.8%. The mixed datasets had intermediate values, mean 56.7%. Pfam domains cover the cores of the domains. This leaves a number of sites in proteins outside the classified regions. Therefore, we cannot even expect all variants to appear in Pfam domains. The KS statistics showed *p*-values < 0.01 for all datasets, so all datasets have non-random biased distributions. The datasets show rather wide distributions to the Pfam families (Table 8). Altogether 14 datasets are mapped to more than 1000 families, and two datasets (DS1 and DS24) to more than 3000 families. The larger datasets cover numerous Pfam families. The coverage of most of the datasets is in the order of 30% or somewhat lower, the largest datasets 1, 21 and 24 being the major exceptions (Table 7).

### Distribution of EC categories in variation datasets

Enzyme activities are classified with EC categories at 4 levels of increasing specificity. 4220 (21%) out of the 20,201 human proteins were allocated to one or more EC classes. At the first level, 4692 proteins could be allocated, at the second level 4605, at the third level 4479 proteins, and 3619 at the fourth level. The reason for these differences is that classifications for some proteins are not complete and do not include all the four levels. The results for the distribution of the datasets to EC classes are in Additional file 3. A summary of the results is in Table 9.

**Table 9:** Mapping of datasets to EC classification at 4 levels

dataset	number of variants with EC numbers	% of total number of variants	KS 1st level	KS 2nd level	KS 3rd level	KS 4th level
DS1	92,063	20.64	0.17 (0.99996)	0.16 (0.41923)	0.15 (0.04553)	0.41 (< 10^− 4^)
DS2	4665	19.71	0.17 (0.99996)	0.15 (0.57158)	0.22 (0.00050)	0.43 (< 10^− 4^)
DS3	7190	37.19	0.33 (0.80956)	0.27 (0.02676)	0.42 (< 10^− 4^)	0.81 (< 10^− 4^)
DS4	4754	24.43	0.17 (0.99996)	0.15 (0.57158)	0.23 (0.00020)	0.44 (< 10^− 4^)
DS5	6951	47.58	0.33 (0.80956)	0.27 (0.02676)	0.43 (< 10^− 4^)	0.82 (< 10^− 4^)
DS6	3911	22.19	0.17 (0.99996)	0.11 (0.88044)	0.16 (0.01740)	0.54 (< 10^− 4^)
DS7	6744	38.48	0.33 (0.80956)	0.27 (0.02676)	0.43 (< 10^− 4^)	0.83 (< 10^− 4^)
DS8	3552	24.25	0.17 (0.99996)	0.13 (0.73544)	0.17 (0.01232)	0.55 (< 10^− 4^)
DS9	6485	49.52	0.33 (0.80956)	0.27 (0.02676)	0.44 (< 10^−4^)	0.83 (< 10^− 4^)
DS10	3035	23.23	0.17 (0.99996)	0.13 (0.73544)	0.18 (0.00596)	0.53 (< 10^−4^)
DS11	6445	51.22	0.33 (0.80956)	0.31 (0.00785)	0.45 (< 10^−4^)	0.83 (< 10^− 4^)
DS12	455	28.35	0.17 (0.99996)	0.73 (< 10^−4^)	0.85 (< 10^− 4^)	0.97 (< 10^− 4^)
DS13	320	24.60	0.50 (0.31803)	0.82 (< 10^−4^)	0.92 (< 10^− 4^)	0.99 (< 10^− 4^)
DS14	1758	20.29	0.17 (0.99996)	0.22 (0.12644)	0.30 (< 10^−4^)	0.65 (< 10^− 4^)
DS15	3880	54.26	0.33 (0.80956)	0.29 (0.01477)	0.44 (< 10^−4^)	0.81 (< 10^− 4^)
DS16	264	25.07	0.50 (0.31803)	0.75 (< 10^−4^)	0.87 (< 10^− 4^)	0.98 (< 10^− 4^)
DS17	207	27.56	0.50 (0.31803)	0.85 (< 10^−4^)	0.93 (< 10^− 4^)	0.99 (< 10^− 4^)
DS18	4585	28.48	0.33 (0.80956)	0.18 (0.29309)	0.29 (< 10^−4^)	0.65 (< 10^− 4^)
DS19	2283	22.24	0.33 (0.80956)	0.20 (0.19638)	0.26 (< 10^−4^)	0.67 (< 10^− 4^)
DS20	3142	35.50	0.50 (0.31803)	0.13 (0.73544)	0.24 (< 10^−4^)	0.64 (< 10^− 4^)
DS21	12,723	31.50	0.33 (0.80956)	0.13 (0.73544)	0.19 (0.00407)	0.60 (< 10^−4^)
DS22	4841	22.89	0.17 (0.99996)	0.11 (0.88044)	0.22 (0.0032)	0.43 (< 10^−4^)
DS23	8710	39.24	0.33 (0.80956)	0.16 (0.41923)	0.35 (< 10^−4^)	0.72 (< 10^− 4^)
DS24	24,218	32.27	0.17 (0.99996)	0.09 (0.97024)	0.16 (0.01740)	0.57 (< 10^−4^)

^a^*p*-value between brackets

The percentages of variants with an EC classification was for the neutral datasets (DS1, DS2, DS4, DS6, DS8, DS10, DS12, DS14, DS16 and DS22) almost always lower than those for the pathogenic datasets (DS3, DS5, DS7, DS9, DS11, DS13, DS15, DS17 and DS23). Again, DS12 and DS13 and their subsets DS16 and DS17 are behaving differently, here the percentages are close to each other (28.4 and 24.6% for DS12 and DS13, respectively, and 25.1 and 27.6% for DS16 and DS17, respectively). Mean value for the neutral datasets without DS12 and DS16 is 20.2%, for the pathogenic datasets without DS13 and DS17 the mean is 45.4%. The mean percentage for the mixed datasets (DS18-DS21 and DS24) was 30.0%, so intermediate, as for the Pfam domains.

On the first level of the EC classification all datasets showed no significant difference in distribution compared to the reference set (Table 9). There are just six categories at the fist level. On the second level, DS11, DS12, DS13, DS16 and DS17 showed biased distributions. When omitting DS11, which had a *p*-value close to 0.01, we see again the distinct character of DS12 and DS13, and their subsets DS16 and DS17. On the third level, most datasets except for DS1, DS6, DS8 and DS25 (*p* > 0.01) are biased, whereas on the 4th level all datasets were significantly different from the distribution for the human proteome. Not all proteins are enzymes, and variants can be located even in proteins that have enzymatic activity outside the catalytic domains. The data for coverage in Table 7 show quite even values up to the second level and decreasing coverage towards the fourth level. UniProt includes practically all the EC categories and DS24 85.5%. The dataset size and EC number coverage have a clear correlation.

### Distribution of GO terms on variation datasets

For further classification of the functions of the proteins in the datasets the GO annotations for each protein were obtained. Mapping of the 20,201 protein-coding genes in the human genome to GO yielded 19,137 UniProt entries (95%) with one or more GO terms (Additional file 4). The frequencies of the unique GO terms were calculated, and served as the reference for testing. In Table 10 the number of unique GO terms found in each dataset and the KS test result on term level and on aspect levels (MF, BP and CC) are shown.

**Table 10:** Number of unique Gene Ontology (GO) terms allocated to each dataset, Kolmogorov-Smirnov 2-sample test statistics (KS) on term level and on GO aspect level (molecular function, cellular component, biological process)

dataset	number of unique GO terms	KS statistic term level	KS statistic aspect level
DS1	17,343	0.27 (< 10^− 4^)^a^	0.33 (0.97621)
DS2	12,869	0.40 (< 10^−4^)	0.33 (0.97621)
DS3	8118	0.62 (< 10^−4^)	0.67 (0.31972)
DS4	12,510	0.29 (< 10^−4^)	0.33 (0.97621)
DS5	7858	0.60 (< 10^−4^)	0.67 (0.31972)
DS6	11,134	0.37 (< 10^−4^)	0.33 (0.97621)
DS7	7515	0.64 (< 10^−4^)	0.67 (0.31972)
DS8	10,893	0.38 (< 10^−4^)	0.33 (0.97621)
DS9	7329	0.62 (< 10^−4^)	0.67 (0.31972)
DS10	11,041	0.37 (< 10^−4^)	0.33 (0.97621)
DS11	7434	0.63 (< 10^−4^)	0.67 (0.31972)
DS12	3194	0.82 (< 10^−4^)	0.33 (0.97621)
DS13	1587	0.91 (< 10^−4^)	0.67 (0.31972)
DS14	9149	0.48 (< 10^−4^)	0.33 (0.97621)
DS15	6739	0.62 (< 10^−4^)	0.67 (0.31972)
DS16	2597	0.85 (< 10^−4^)	0.33 (0.97621)
DS17	1459	0.92 (< 10^−4^)	0.67 (0.31972)
DS18	10,345	0.54 (< 10^−4^)	0.33 (0.97621)
DS19	10,393	0.58 (< 10^−4^)	0.67 (0.31972)
DS20	10,468	0.41 (< 10^−4^)	0.33 (0.97621)
DS21	14,492	0.41 (< 10^−4^)	0.33 (0.97621)
DS22	13,318	0.38 (< 10^−4^)	0.33 (0.97621)
DS23	9739	0.54 (< 10^−4^)	0.67 (0.31972)
DS24	16,180	0.36 (< 10^−4^)	0.33 (0.97621)

^a^*p*-value between brackets

On the aspect level of the GO, no dataset had a significantly different distribution when compared to the reference set (Table 10**)**. On term level, all datasets had a significantly different distribution when compared to the reference set. On aspect level, the KS statistic and *p*-values were all 0.33 and 0.97621, respectively, for all neutral datasets, and 0.67 and 0.31972, respectively, for all pathogenic datasets. For the mixed datasets, these values were the same as for the neutral datasets, except for DS19.

Proteins in 12 of the datasets were mapped to more than 10,000 unique GO terms, while the total number for the entire human proteome is 17,637. Although the datasets contain thousands of GO annotations, they are far from being fully representative. On the other hand, for that the datasets should be rather large due to the size of the GO. Still, the GO coverage is clearly higher than for the other functional and structural classifications except for the first two levels in CATH and EC (Table 7).

## Discussion

ML methods are used to generalize from the training data to unknown ones. If training is done on unrepresentative data, the method cannot learn all features of the event space and will be biased. Similarly, when testing method performance, the test data should cover the space to assess the performance in a realistic way. This is to our knowledge the first study that addresses the variant benchmark dataset representativeness.

The distribution of variants per protein varies greatly which is a result of some proteins/genes and diseases being studied extensively. Therefore, some of the proteins can include more than 2200 variants, whereas others are represented by only a single one. Comparison to ExAC data revealed that all the pathogenic datasets contained a small number of likely benign variants; however, the proportion is so small, < 2%, that it will not have a major effect on the performance or assessment of methods.

To perform the analysis, we first considered what aspects of representativeness are the most relevant for our datasets. We decided to study how representative the datasets are in describing the protein universe in protein fold, domain, enzyme classification, and GO annotation levels as well as for the distribution of the coding genes to chromosomes. As our knowledge of many aspects of the protein universe is limited, we concentrated on the available data and annotations. Only the enzyme classification data were (almost) complete. It is possible that still some new enzymatic activities will be found for human proteins e.g. due to moonlighting/multitasking [42]. Certain characteristics, such as protein structures, are available only for some proteins. In these cases, we collected the current proteome-wide knowledge of the feature and used it as the background for statistical tests.

The distribution tests for CATH, Pfam, EC, and GO data could only be made for a fraction of the variants in the datasets. The mapping to CATH domains depends on mapping to a PDB structure, which in its turn is dependent on the availability of a UniProt protein sequence. In DS1, the largest dataset, 85% of the variants could be mapped to a UniProt sequence, but only 8.8% of the variants could be mapped to a PDB structure, and of these, about 56% had a CATH classification, i.e. less than 5% of the total number of variants in the dataset. For other datasets, the situation was better, e.g. in DS15, 52% of the variants could be mapped to a PDB structure, and of these 67% had a CATH classification, almost 35% of the total number of variants in the dataset. CATH, Pfam, EC and GO annotations may apply only to a part of a protein, therefore we cannot even expect all the variants to fall into these classes.

Suitable statistical tests were chosen to investigate the dataset representativeness. We used the non-parametric Kolmogorov-Smirnov test to compare the dataset distributions to proteome-wide background data. The binomial test was used for the analysis of chromosome distributions. The coverage was calculated based on the numbers of instances in the dataset with a certain classification compared to the background.

Analysis of the chromosomal distribution of variants in the datasets showed that some chromosomes in all the datasets had normal distribution; however, these chromosomes were different for the different datasets. The numbers of variants per chromosome were weighted by the number of genes per chromosome. The differences in the chromosomal distributions largely originate from the uneven distribution of variants to the investigated proteins.

Many of the tested datasets are subsets of larger ones and therefore have related properties. The DS16 and DS17 are subsets of DS12 and DS13, all being small and therefore standing out in many of the statistical tests. The results in Table 11 show that all the datasets have statistically significant deviations from the background distributions at many levels. The space of variants is huge when we consider all the different characteristics, it is thus obvious that small datasets cannot be representative. DS1, which is the largest one with 446,013 variants, shows the highest coverage of included categories in CATH, Pfam, EC and GO, still many of the tests show biased distributions in this dataset. The size is not the only parameter that defines dataset representativeness. The cases should be widely spread into the protein universe.

**Table 11:** Summary of all the test results

dataset	no. of chromosomes^a^	CATH Class level	CATH Architecture level	CATH Topology level	CATH Homology level	EC 1st level	EC 2nd level	EC 3rd level	EC 4th level	Pfam	GO terms level	GO aspect level	score without chromosomes^b^
DS1	7	1^c^	1	0^d^	0	1	1	1	0	0	0	1	6
DS2	8	1	1	0	0	1	1	1	0	0	0	1	6
DS3	3	1	1	0	0	1	1	0	0	0	0	1	5
DS4	9	1	1	0	0	1	1	1	0	0	0	1	6
DS5	5	1	1	0	0	1	1	0	0	0	0	1	5
DS6	9	1	1	0	0	1	1	1	0	0	0	1	6
DS7	3	1	1	0	0	1	1	0	0	0	0	1	5
DS8	7	1	1	0	0	1	1	1	0	0	0	1	6
DS9	6	1	1	0	0	1	1	0	0	0	0	1	5
DS10	9	1	1	0	0	1	1	1	0	0	0	1	6
DS11	5	1	1	0	0	1	1	0	0	0	0	1	5
DS12	9	1	0	0	0	1	0	0	0	0	0	1	3
DS13	3	1	0	0	0	1	0	0	0	0	0	1	3
DS14	11	1	1	0	0	1	1	0	0	0	0	1	5
DS15	5	1	1	0	0	1	1	0	0	0	0	1	5
DS16	13	1	0	0	0	1	0	0	0	0	0	1	3
DS17	6	1	0	0	0	1	0	0	0	0	0	1	3
DS18	6	1	1	0	0	1	1	0	0	0	0	1	5
DS19	5	1	1	0	0	1	1	0	0	0	0	1	5
DS20	11	1	1	0	0	1	1	0	0	0	0	1	5
DS21	4	1	1	0	0	1	1	1	0	0	0	1	6
DS22	11	1	1	0	0	1	1	1	0	0	0	1	6
DS23	3	1	1	0	0	1	1	0	0	0	0	1	5
DS24	2	1	1	0	0	1	1	1	0	0	0	1	6

^a^number of chromosomes with unbiased distribution of variants

^b^sum of scores in all categories tested

^c^category has score 1 if distribution was unbiased

^d^category has score 0 if distribution was biased

The results show that all the datasets are more or less unrepresentative of the protein universe. The space of the variants and effects is huge and therefore the current datasets cannot be fully representative. When we are looking at the coverage to the investigated categories, the situation looks more encouraging. Most of the datasets display a wide coverage of categories. The major reason for this is the still limited number of verified cases. Many datasets have included practically all available cases without having a chance to set further requirements. As the experimental data is highly biased and certain diseases are well studied and contain large numbers of variants, the distribution to the character space is therefore uneven.

For some features, especially at the higher levels of the CATH and EC hierarchies, and the GO annotation at the aspect level, all datasets were found to be unbiased. For other features, no one dataset was found unbiased. These features were CATH at the Topology and Homology level, EC at the 4th level, Pfam and GO at the terms level.

ML methods are trained to generalize based on the given examples. Reliable, high-quality and representative datasets are essential for this. Evaluation of the effect of the lack of representativeness on ML method performance is difficult. This is because, in addition to the dataset and its qualities, many other factors contribute, including how the ML method is trained, tested, implemented, which features are used and how they have been selected. Further, other aspects of the datasets in addition to representativeness also contribute to the predictor performance. We recently addressed the relevance of SAAS data for stability prediction [43].

The VariBench database contains training datasets that have been used for several tolerance predictors. There are datasets both for PON-P [18] (DS4 and DS5) and PON-P2 [13] (DS10 and DS11). The SwissVar dataset (DS24) and HumVar selections (DS22 and DS23) have been used several times, including MetaLR and MetaSVM [17], MutationTaster2 [11], PolyPhen-2 [12], PROVEAN [7] and SNP&GO [44]. The performances of these tools have been assessed several times and with different test datasets, many of which were included to the analyses [12, 13, 28–30]. MetaLR, MetaSVM and PON-P2 have been among the best tools.

The datasets used for training the predictors do not show clear correlation between representativeness and performance. The PON-P2 training sets are smaller than those based on SwissVar. Similarly, the coverage of the PON-P2 datasets is smaller than for the SwissVar datasets on all the investigated features. Representativeness is but one of the features for benchmarks [1]. SwissVar, which is the second largest dataset, contains in addition to disease-causing variants in Mendelian disorders also variants that have been identified in complex diseases including cancers. Tests for the relevance of these variants in diseases are usually missing. Recently it was shown that only 14% of the variants in COSMIC database [45] are likely harmful [46]. Therefore, datasets based on SwissVar likely contain benign variants, which have a detrimental effect on the performance of methods trained on these datasets. These variants have been filtered away from the PON-P and PON-P2 datasets, which could partly describe why these tools have better performance despite smaller training datasets. This implies the importance of the benchmark relevance criterion.

Although the best methods trained with the tested datasets have high performance, it is likely that more representative datasets would improve their performance. There are two areas where major improvements would be expected. First, variants of unknown significance could be classified more reliably. However, it is important to notice that there are not just two extremes, there is indeed a continuum of pathogenicity [39]. Another area where better representativeness would have an impact is in the performance on hard to predict cases [47], especially when dealing with sequences with a small number of related ones or unique human proteins. The independent test sets (DS12 and DS13, and their derivatives DS16 and DS17) used in method development, are very small and therefore not very representative regarding the proteome properties. This problem can only be overcome by generating larger high-quality datasets.

## Conclusions

The analysis revealed that none of the available variant datasets is fully representative. The larger datasets are typically better with higher coverage. Datasets for neutral variants are better than the pathogenic datasets. Despite the lack of representativeness, many datasets cover a large number of the categories in the investigated features. Correlation was not observed between the dataset representativeness and the performance of methods trained on them. Several additional features are of importance as well. High-quality benchmark datasets are expensive to produce, and the amount of available verified cases is still limited. We suggest that in the future method developers and assessors should take the dataset representativeness into account. It would likely improve performance especially in the prediction of variants in difficult, even unique genes and proteins, as well as help in further grouping of unclassified variants.

## Additional files

Additional file 1: **Tables S1-S24** with the chromosomal distributions of variants in datasets DS2-DS24. (PDF 122 kb)

Additional file 2: Reference data for 5734 Pfam domains and their frequencies in the entire human proteome. (TSV 56 kb)

Additional file 3: Distribution of the datasets to EC classes. (XLSX 192 kb)

Additional file 4: Mapping of 19,137 UniProt entries to GO terms. (TSV 8810 kb)

## Abbreviations

CDS: Coding sequence
EC: Enzyme Commission
GO: Gene Ontology
MAF: Minor allele frequency
ML: Machine learning
RSCB: Research Collaboratory for Structural Bioinformatics
SAAS: Single amino acid substitution
SNV: Single nucleotide variant
