# Supplementary material for: Representativeness of variation benchmark datasets
Source: BMC Bioinformatics. 2018 Nov 29;19:461. doi: 10.1186/s12859-018-2478-6 (PMC6267811; doi:10.1186/s12859-018-2478-6)
Supplement: Supplementary file 1 — Tables S1-S24 with the chromosomal distributions of variants in datasets DS2-DS24. (PDF 122 kb) [file 12859_2018_2478_MOESM1_ESM.pdf]

Table S1: Chromosomal distribution of variants in dataset DS2

chromosome	no. of genes	CDS length	no. of observed variants	no. of expected variants (no. of genes)	no. of expected variants (CDS length)	p-value[a] (no. of genes)	p-value[a] (CDS length)
1	2037	3483903	2444	2437	2406	0.880988	0.41992
2	1238	2517642	1662	1481	1739	$<10^{-4}$	0.055072
3	1071	1965098	1381	1281	1357	0.004449	0.502264
4	745	1365661	1012	891	943	$<10^{-4}$	0.02383
5	882	1601648	1256	1055	1106	$<10^{-4}$	$<10^{-4}$
6	1035	1735760	1510	1238	1199	$<10^{-4}$	$<10^{-4}$
7	901	1609177	1387	1078	1111	$<10^{-4}$	$<10^{-4}$
8	668	1135640	790	799	784	0.759678	0.827537
9	770	1382150	922	921	955	0.973185	0.290376
10	727	1322286	1006	870	913	$<10^{-4}$	0.002013
11	1278	2005315	1534	1529	1385	0.894814	$<10^{-4}$
12	1033	1776908	1217	1236	1227	0.588847	0.780629
13	324	634435	391	388	438	0.877928	0.02198
14	614	1079560	873	735	746	$<10^{-4}$	$<10^{-4}$
15	589	1189858	722	705	822	0.515641	0.000334
16	858	1451775	922	1026	1003	0.000802	0.008941
17	1184	1971211	1153	1416	1362	$<10^{-4}$	$<10^{-4}$
18	268	534152	410	321	369	$<10^{-4}$	0.033517
19	1467	2277812	1470	1755	1573	$<10^{-4}$	0.006914
20	540	811690	641	646	561	0.85754	0.000732
21	233	342226	193	279	236	$<10^{-4}$	0.003999
22	439	712404	461	525	492	0.004403	0.164606
X	840	1296174	306	1005	895	$<10^{-4}$	$<10^{-4}$
Y	45	67500	8	54	47	$<10^{-4}$	$<10^{-4}$

[a]results of binomial test

Table S2: Chromosomal distribution of variants in dataset DS3

chromosome	no. of genes	CDS length	no. of observed variants	no. of expected variants (no. of genes)	no. of expected variants (CDS length)	p-value[a] (no. of genes)	p-value[a] (CDS length)
1	2037	3483903	1729	1975	1950	$<10^{-4}$	$<10^{-4}$
2	1238	2517642	843	1200	1409	$<10^{-4}$	$<10^{-4}$
3	1071	1965098	891	1038	1100	$<10^{-4}$	$<10^{-4}$
4	745	1365661	209	722	764	$<10^{-4}$	$<10^{-4}$
5	882	1601648	355	855	897	$<10^{-4}$	$<10^{-4}$
6	1035	1735760	389	1004	972	$<10^{-4}$	$<10^{-4}$
7	901	1609177	716	874	901	$<10^{-4}$	$<10^{-4}$
8	668	1135640	226	648	636	$<10^{-4}$	$<10^{-4}$
9	770	1382150	422	747	774	$<10^{-4}$	$<10^{-4}$
10	727	1322286	470	705	740	$<10^{-4}$	$<10^{-4}$
11	1278	2005315	1518	1239	1123	$<10^{-4}$	$<10^{-4}$
12	1033	1776908	1058	1002	995	0.071673	0.040213
13	324	634435	258	314	355	0.001173	$<10^{-4}$
14	614	1079560	596	595	604	0.966776	0.756528
15	589	1189858	634	571	666	0.008416	0.214072
16	858	1451775	838	832	813	0.831566	0.360673
17	1184	1971211	3345	1148	1103	$<10^{-4}$	$<10^{-4}$
18	268	534152	153	260	299	$<10^{-4}$	$<10^{-4}$
19	1467	2277812	634	1422	1275	$<10^{-4}$	$<10^{-4}$
20	540	811690	326	524	454	$<10^{-4}$	$<10^{-4}$
21	233	342226	289	226	192	$<10^{-4}$	$<10^{-4}$
22	439	712404	108	426	399	$<10^{-4}$	$<10^{-4}$
X	840	1296174	3151	814	726	$<10^{-4}$	$<10^{-4}$
Y	45	67500	26	44	38	0.005055	0.060258

[a]results of binomial test

Table S3: Chromosomal distribution of variants in dataset DS4

chromosome	no. of genes	CDS length	no. of observed variants	[illegible] variants (no. of genes)	[illegible] variants (CDS length)	p-value[a] (no. of genes)	p-value[a] (CDS length)
1	2037	3483903	1939	2003	1978	0.134108	0.36108
2	1238	2517642	1398	1218	1430	$<10^{-4}$	0.394322
3	1071	1965098	1205	1053	1116	$<10^{-4}$	0.006353
4	745	1365661	857	733	775	$<10^{-4}$	0.00317
5	882	1601648	977	867	909	0.000174	0.022858
6	1035	1735760	1227	1018	986	$<10^{-4}$	$<10^{-4}$
7	901	1609177	989	886	914	0.000481	0.01157
8	668	1135640	692	657	645	0.164764	0.059779
9	770	1382150	740	757	785	0.540728	0.104875
10	727	1322286	839	715	751	$<10^{-4}$	0.001201
11	1278	2005315	1256	1257	1139	0.988366	0.000418
12	1033	1776908	1011	1016	1009	0.884704	0.948439
13	324	634435	342	319	360	0.194052	0.351908
14	614	1079560	682	604	613	0.00156	0.005245
15	589	1189858	562	579	676	0.486333	$<10^{-4}$
16	858	1451775	798	844	824	0.109269	0.354793
17	1184	1971211	969	1164	1119	$<10^{-4}$	$<10^{-4}$
18	268	534152	333	264	303	$<10^{-4}$	0.087692
19	1467	2277812	1249	1443	1293	$<10^{-4}$	0.205406
20	540	811690	575	531	461	0.055559	$<10^{-4}$
21	233	342226	156	229	194	$<10^{-4}$	0.004899
22	439	712404	382	432	405	0.014945	0.268921
X	840	1296174	274	826	736	$<10^{-4}$	$<10^{-4}$
Y	45	67500	7	44	38	$<10^{-4}$	$<10^{-4}$

[a]results of binomial test

Table S4: Chromosomal distribution of variants in dataset DS5

chromosome	no. of genes	CDS length	no. of observed variants	no. of expected variants (no. of genes)	no. of expected variants (CDS length)	p-value[a] (no. of genes)	p-value[a] (CDS length)
1	2037	3483903	1594	1490	1471	0.004837	0.000829
2	1238	2517642	608	905	1063	$<10^{-4}$	$<10^{-4}$
3	1071	1965098	632	783	830	$<10^{-4}$	$<10^{-4}$
4	745	1365661	201	545	577	$<10^{-4}$	$<10^{-4}$
5	882	1601648	352	645	676	$<10^{-4}$	$<10^{-4}$
6	1035	1735760	359	757	733	$<10^{-4}$	$<10^{-4}$
7	901	1609177	675	659	679	0.523451	0.890589
8	668	1135640	212	489	480	$<10^{-4}$	$<10^{-4}$
9	770	1382150	408	563	584	$<10^{-4}$	$<10^{-4}$
10	727	1322286	439	532	558	$<10^{-4}$	$<10^{-4}$
11	1278	2005315	1267	935	847	$<10^{-4}$	$<10^{-4}$
12	1033	1776908	944	755	750	$<10^{-4}$	$<10^{-4}$
13	324	634435	255	237	268	0.238294	0.459105
14	614	1079560	400	449	456	0.018774	0.007667
15	589	1189858	424	431	502	0.750606	0.000304
16	858	1451775	799	627	613	$<10^{-4}$	$<10^{-4}$
17	1184	1971211	912	866	832	0.106933	0.004788
18	268	534152	153	196	226	0.001541	$<10^{-4}$
19	1467	2277812	557	1073	962	$<10^{-4}$	$<10^{-4}$
20	540	811690	296	395	343	$<10^{-4}$	0.010153
21	233	342226	284	170	144	$<10^{-4}$	$<10^{-4}$
22	439	712404	92	321	301	$<10^{-4}$	$<10^{-4}$
X	840	1296174	2581	614	547	$<10^{-4}$	$<10^{-4}$
Y	45	67500	26	33	29	0.256435	0.707943

[a]results of binomial test

Table S5: Chromosomal distribution of variants in dataset DS6

chromosome	no. of genes	CDS length	no. of observed variants	no. of expected variants (no. of genes)	no. of expected variants (CDS length)	p-value[a] (no. of genes)	p-value[a] (CDS length)
1	2037	3483903	1816	1813	1791	0.940698	0.524932
2	1238	2517642	1308	1102	1294	$<10^{-4}$	0.685974
3	1071	1965098	1038	953	1010	0.005136	0.364168
4	745	1365661	834	663	702	$<10^{-4}$	$<10^{-4}$
5	882	1601648	943	785	823	$<10^{-4}$	$<10^{-4}$
6	1035	1735760	1115	921	892	$<10^{-4}$	$<10^{-4}$
7	901	1609177	1097	802	827	$<10^{-4}$	$<10^{-4}$
8	668	1135640	615	595	584	0.404134	0.191855
9	770	1382150	719	686	710	0.198662	0.730353
10	727	1322286	792	647	680	$<10^{-4}$	$<10^{-4}$
11	1278	2005315	1013	1138	1031	$<10^{-4}$	0.585241
12	1033	1776908	895	920	913	0.406699	0.552027
13	324	634435	319	288	326	0.06984	0.716352
14	614	1079560	718	547	555	$<10^{-4}$	$<10^{-4}$
15	589	1189858	568	524	612	0.053639	0.073379
16	858	1451775	738	764	746	0.345536	0.779035
17	1184	1971211	798	1054	1013	$<10^{-4}$	$<10^{-4}$
18	268	534152	299	239	275	$<10^{-4}$	0.136135
19	1467	2277812	823	1306	1171	$<10^{-4}$	$<10^{-4}$
20	540	811690	490	481	417	0.677291	0.000432
21	233	342226	146	207	176	$<10^{-4}$	0.022909
22	439	712404	386	391	366	0.817997	0.290854
X	840	1296174	138	748	666	$<10^{-4}$	$<10^{-4}$
Y	45	67500	7	40	35	$<10^{-4}$	$<10^{-4}$

[a]results of binomial test

Table S6: Chromosomal distribution of variants in dataset DS7

chromosome	no. of genes	CDS length	no. of observed variants	no. of expected variants (no. of genes)	no. of expected variants (CDS length)	p-value[a] (no. of genes)	p-value[a] (CDS length)
1	2037	3483903	1651	1790	1767	0.000475	0.003452
2	1238	2517642	642	1088	1277	$<10^{-4}$	$<10^{-4}$
3	1071	1965098	788	941	997	$<10^{-4}$	$<10^{-4}$
4	745	1365661	182	654	693	$<10^{-4}$	$<10^{-4}$
5	882	1601648	267	775	812	$<10^{-4}$	$<10^{-4}$
6	1035	1735760	337	909	880	$<10^{-4}$	$<10^{-4}$
7	901	1609177	570	792	816	$<10^{-4}$	$<10^{-4}$
8	668	1135640	144	587	576	$<10^{-4}$	$<10^{-4}$
9	770	1382150	391	676	701	$<10^{-4}$	$<10^{-4}$
10	727	1322286	460	639	671	$<10^{-4}$	$<10^{-4}$
11	1278	2005315	1335	1123	1017	$<10^{-4}$	$<10^{-4}$
12	1033	1776908	875	907	901	0.282639	0.382993
13	324	634435	211	285	322	$<10^{-4}$	$<10^{-4}$
14	614	1079560	560	539	548	0.358069	0.587218
15	589	1189858	621	517	604	$<10^{-4}$	0.468365
16	858	1451775	716	754	736	0.162572	0.4627
17	1184	1971211	3104	1040	1000	$<10^{-4}$	$<10^{-4}$
18	268	534152	148	235	271	$<10^{-4}$	$<10^{-4}$
19	1467	2277812	609	1289	1155	$<10^{-4}$	$<10^{-4}$
20	540	811690	314	474	412	$<10^{-4}$	$<10^{-4}$
21	233	342226	272	205	174	$<10^{-4}$	$<10^{-4}$
22	439	712404	82	386	361	$<10^{-4}$	$<10^{-4}$
X	840	1296174	3077	738	657	$<10^{-4}$	$<10^{-4}$
Y	45	67500	26	40	34	0.026158	0.171041

[a]results of binomial test

Table S7: Chromosomal distribution of variants in dataset DS8

chromosome	no. of genes	CDS length	no. of observed variants	no. of expected variants (no. of genes)	no. of expected variants (CDS length)	p-value[a] (no. of genes)	p-value[a] (CDS length)
1	2037	3483903	1441	1507	1488	0.074809	0.198659
2	1238	2517642	1113	916	1076	$<10^{-4}$	0.234847
3	1071	1965098	957	792	839	$<10^{-4}$	$<10^{-4}$
4	745	1365661	722	551	583	$<10^{-4}$	$<10^{-4}$
5	882	1601648	669	653	684	0.521736	0.570168
6	1035	1735760	978	766	742	$<10^{-4}$	$<10^{-4}$
7	901	1609177	762	667	687	0.000226	0.004075
8	668	1135640	562	494	485	0.002159	0.000531
9	770	1382150	595	570	590	0.285382	0.833646
10	727	1322286	673	538	565	$<10^{-4}$	$<10^{-4}$
11	1278	2005315	837	946	857	0.000203	0.503456
12	1033	1776908	775	764	759	0.682668	0.550883
13	324	634435	282	240	271	0.007574	0.49991
14	614	1079560	539	454	461	$<10^{-4}$	0.000321
15	589	1189858	447	436	508	0.592691	0.005118
16	858	1451775	644	635	620	0.714951	0.324717
17	1184	1971211	671	876	842	$<10^{-4}$	$<10^{-4}$
18	268	534152	231	198	228	0.01999	0.841313
19	1467	2277812	698	1085	973	0	$<10^{-4}$
20	540	811690	461	400	347	0.002344	$<10^{-4}$
21	233	342226	123	172	146	$<10^{-4}$	0.055714
22	439	712404	334	325	304	0.613555	0.087408
X	840	1296174	120	622	554	$<10^{-4}$	$<10^{-4}$
Y	45	67500	6	33	29	$<10^{-4}$	$<10^{-4}$

[a]results of binomial test

Table S8: Chromosomal distribution of variants in dataset DS9

chromosome	no. of genes	CDS length	no. of observed variants	no. of expected variants (no. of genes)	no. of expected variants (CDS length)	p-value[a] (no. of genes)	p-value[a] (CDS length)
1	2037	3483903	1536	1334	1317	$<10^{-4}$	$<10^{-4}$
2	1238	2517642	390	811	952	$<10^{-4}$	$<10^{-4}$
3	1071	1965098	565	701	743	$<10^{-4}$	$<10^{-4}$
4	745	1365661	177	488	516	$<10^{-4}$	$<10^{-4}$
5	882	1601648	264	578	606	$<10^{-4}$	$<10^{-4}$
6	1035	1735760	314	678	656	$<10^{-4}$	$<10^{-4}$
7	901	1609177	580	590	608	0.688927	0.24489
8	668	1135640	138	437	429	$<10^{-4}$	$<10^{-4}$
9	770	1382150	380	504	523	$<10^{-4}$	$<10^{-4}$
10	727	1322286	429	476	500	0.028128	0.001019
11	1278	2005315	1087	837	758	$<10^{-4}$	$<10^{-4}$
12	1033	1776908	816	677	672	$<10^{-4}$	$<10^{-4}$
13	324	634435	210	212	240	0.917285	0.050553
14	614	1079560	366	402	408	0.068133	0.03459
15	589	1189858	411	386	450	0.196344	0.064598
16	858	1451775	688	562	549	$<10^{-4}$	$<10^{-4}$
17	1184	1971211	805	775	745	0.266372	0.025976
18	268	534152	148	176	202	0.033469	$<10^{-4}$
19	1467	2277812	533	961	861	$<10^{-4}$	$<10^{-4}$
20	540	811690	284	354	307	$<10^{-4}$	$<10^{-4}$
21	233	342226	270	153	129	$<10^{-4}$	$<10^{-4}$
22	439	712404	82	288	269	$<10^{-4}$	$<10^{-4}$
X	840	1296174	2459	550	490	$<10^{-4}$	$<10^{-4}$
Y	45	67500	26	29	26	0.64236	0.920826

[a]results of binomial test

Table S9: Chromosomal distribution of variants in dataset DS10

chromosome	no. of genes	CDS length	no. of observed variants	no. of expected variants (no. of genes)	no. of expected variants (CDS length)	p-value[a] (no. of genes)	p-value[a] (CDS length)
1	2037	3483903	1427	1345	1328	0.018951	0.004544
2	1238	2517642	886	817	960	0.013312	0.01307
3	1071	1965098	788	707	749	0.001975	0.142191
4	745	1365661	605	492	521	$<10^{-4}$	0.000222
5	882	1601648	636	582	611	0.02326	0.290454
6	1035	1735760	715	683	662	0.208444	0.034434
7	901	1609177	613	595	613	0.449999	1
8	668	1135640	476	441	433	0.089977	0.037704
9	770	1382150	542	508	527	0.123855	0.490641
10	727	1322286	551	480	504	0.001222	0.034616
11	1278	2005315	894	844	764	0.078076	$<10^{-4}$
12	1033	1776908	676	682	677	0.82874	0.984257
13	324	634435	243	214	242	0.049354	0.922434
14	614	1079560	454	405	412	0.014344	0.035342
15	589	1189858	386	389	454	0.897617	0.001056
16	858	1451775	593	566	553	0.245874	0.086164
17	1184	1971211	626	782	751	$<10^{-4}$	$<10^{-4}$
18	268	534152	209	177	204	0.01709	0.697557
19	1467	2277812	851	969	868	$<10^{-4}$	0.562188
20	540	811690	395	357	309	0.044106	$<10^{-4}$
21	233	342226	113	154	130	$<10^{-4}$	0.134386
22	439	712404	265	290	272	0.145553	0.712918
X	840	1296174	119	555	494	$<10^{-4}$	$<10^{-4}$
Y	45	67500	0	30	26	$<10^{-4}$	$<10^{-4}$

[a]results of binomial test

Table S10: Chromosomal distribution of variants in dataset DS11

chromosome	no. of genes	CDS length	no. of observed variants	no. of expected variants (no. of genes)	no. of expected variants (CDS length)	p-value[a] (no. of genes)	p-value[a] (CDS length)
1	2037	3483903	1525	1282	1266	$<10^{-4}$	$<10^{-4}$
2	1238	2517642	510	779	915	$<10^{-4}$	$<10^{-4}$
3	1071	1965098	538	674	714	$<10^{-4}$	$<10^{-4}$
4	745	1365661	184	469	496	$<10^{-4}$	$<10^{-4}$
5	882	1601648	316	555	582	$<10^{-4}$	$<10^{-4}$
6	1035	1735760	252	651	631	$<10^{-4}$	$<10^{-4}$
7	901	1609177	499	567	585	0.003223	0.000227
8	668	1135640	174	420	413	$<10^{-4}$	$<10^{-4}$
9	770	1382150	326	485	502	$<10^{-4}$	$<10^{-4}$
10	727	1322286	420	457	480	0.081834	0.004521
11	1278	2005315	1078	804	729	$<10^{-4}$	$<10^{-4}$
12	1033	1776908	806	650	646	$<10^{-4}$	$<10^{-4}$
13	324	634435	236	204	230	0.026083	0.714501
14	614	1079560	334	386	392	0.006616	0.002458
15	589	1189858	373	371	432	0.916025	0.003301
16	858	1451775	571	540	527	0.172526	0.052908
17	1184	1971211	707	745	716	0.156442	0.743522
18	268	534152	141	169	194	0.029989	$<10^{-4}$
19	1467	2277812	526	923	828	$<10^{-4}$	$<10^{-4}$
20	540	811690	271	340	295	0.000104	0.165959
21	233	342226	239	147	124	$<10^{-4}$	$<10^{-4}$
22	439	712404	90	276	259	$<10^{-4}$	$<10^{-4}$
X	840	1296174	2308	529	471	$<10^{-4}$	$<10^{-4}$
Y	45	67500	26	28	25	0.77684	0.760934

[a]results of binomial test

Table S11: Chromosomal distribution of variants in dataset DS12

chromosome	no. of genes	CDS length	no. of observed variants	no. of expected variants (no. of genes)	no. of expected variants (CDS length)	p-value[a] (no. of genes)	p-value[a] (CDS length)
1	2037	3483903	152	165	163	0.304154	0.385548
2	1238	2517642	126	100	118	0.009703	0.44363
3	1071	1965098	121	87	92	0.000332	0.002598
4	745	1365661	72	60	64	0.114235	0.306567
5	882	1601648	117	72	75	$<10^{-4}$	$<10^{-4}$
6	1035	1735760	128	84	81	$<10^{-4}$	$<10^{-4}$
7	901	1609177	85	73	75	0.150368	0.261614
8	668	1135640	75	54	53	0.005485	0.004098
9	770	1382150	72	62	65	0.19486	0.341066
10	727	1322286	83	59	62	0.002244	0.009316
11	1278	2005315	73	104	94	0.001156	0.025356
12	1033	1776908	63	84	83	0.018378	0.020923
13	324	634435	24	26	30	0.767078	0.353086
14	614	1079560	65	50	51	0.036779	0.044863
15	589	1189858	62	48	56	0.047316	0.375146
16	858	1451775	44	70	68	0.000935	0.002313
17	1184	1971211	49	96	92	$<10^{-4}$	$<10^{-4}$
18	268	534152	25	22	25	0.517996	1
19	1467	2277812	68	119	107	$<10^{-4}$	$<10^{-4}$
20	540	811690	34	44	38	0.145554	0.565668
21	233	342226	17	19	16	0.729639	0.800745
22	439	712404	25	36	33	0.063458	0.160836
X	840	1296174	25	68	61	$<10^{-4}$	$<10^{-4}$
Y	45	67500	0	4	3	0.03944	0.084349

[a]results of binomial test

Table S12: Chromosomal distribution of variants in dataset DS13

chromosome	no. of genes	CDS length	no. of observed variants	no. of expected variants (no. of genes)	no. of expected variants (CDS length)	p-value[a] (no. of genes)	p-value[a] (CDS length)
1	2037	3483903	44	134	132	$<10^{-4}$	$<10^{-4}$
2	1238	2517642	82	81	95	0.908536	0.166409
3	1071	1965098	89	70	74	0.022734	$<10^{-4}$
4	745	1365661	14	49	52	$<10^{-4}$	$<10^{-4}$
5	882	1601648	26	58	61	$<10^{-4}$	$<10^{-4}$
6	1035	1735760	33	68	66	$<10^{-4}$	$<10^{-4}$
7	901	1609177	112	59	61	$<10^{-4}$	$<10^{-4}$
8	668	1135640	8	44	43	$<10^{-4}$	$<10^{-4}$
9	770	1382150	22	50	52	$<10^{-4}$	$<10^{-4}$
10	727	1322286	7	48	50	$<10^{-4}$	$<10^{-4}$
11	1278	2005315	102	84	76	0.047914	0.003032
12	1033	1776908	115	68	67	$<10^{-4}$	$<10^{-4}$
13	324	634435	10	21	24	0.011098	0.001882
14	614	1079560	54	40	41	0.029598	0.046184
15	589	1189858	47	39	45	0.192455	0.761264
16	858	1451775	105	56	55	$<10^{-4}$	$<10^{-4}$
17	1184	1971211	161	78	75	$<10^{-4}$	$<10^{-4}$
18	268	534152	9	18	20	0.031469	0.009337
19	1467	2277812	14	96	86	$<10^{-4}$	$<10^{-4}$
20	540	811690	17	35	31	0.001055	0.010187
21	233	342226	28	15	13	0.002413	0.000208
22	439	712404	2	29	27	$<10^{-4}$	$<10^{-4}$
X	840	1296174	196	55	49	$<10^{-4}$	$<10^{-4}$
Y	45	67500	0	3	3	0.082947	0.193569

[a]results of binomial test

Table S13: Chromosomal distribution of variants in dataset DS14

chromosome	no. of genes	CDS length	no. of observed variants	no. of expected variants (no. of genes)	no. of expected variants (CDS length)	p-value[a] (no. of genes)	p-value[a] (CDS length)
1	2037	3483903	951	892	880	0.038605	0.012795
2	1238	2517642	621	542	636	0.000583	0.550326
3	1071	1965098	537	469	497	0.001584	0.064428
4	745	1365661	397	326	345	$<10^{-4}$	0.005066
5	882	1601648	431	386	405	0.020464	0.185508
6	1035	1735760	484	453	439	0.134592	0.027418
7	901	1609177	402	394	407	0.679901	0.83898
8	668	1135640	312	292	287	0.233692	0.133342
9	770	1382150	374	337	349	0.042474	0.180709
10	727	1322286	381	318	334	0.000439	0.010239
11	1278	2005315	572	559	507	0.569658	0.003376
12	1033	1776908	422	452	449	0.154004	0.198954
13	324	634435	136	142	160	0.641709	0.055519
14	614	1079560	294	269	273	0.121465	0.196222
15	589	1189858	254	258	301	0.824945	0.005771
16	858	1451775	372	376	367	0.853597	0.769203
17	1184	1971211	370	518	498	$<10^{-4}$	$<10^{-4}$
18	268	534152	149	117	135	0.003868	0.224302
19	1467	2277812	590	642	576	0.032912	0.5316
20	540	811690	266	236	205	0.051413	$<10^{-4}$
21	233	342226	58	102	86	$<10^{-4}$	0.001401
22	439	712404	193	192	180	0.941797	0.327326
X	840	1296174	95	368	328	$<10^{-4}$	$<10^{-4}$
Y	45	67500	0	20	17	$<10^{-4}$	$<10^{-4}$

[a]results of binomial test

Table S14: Chromosomal distribution of variants in dataset DS15

chromosome	no. of genes	CDS length	no. of observed variants	no. of expected variants (no. of genes)	no. of expected variants (CDS length)	p-value[a] (no. of genes)	p-value[a] (CDS length)
1	2037	3483903	810	728	719	0.001521	0.000426
2	1238	2517642	244	442	519	$<10^{-4}$	$<10^{-4}$
3	1071	1965098	264	383	405	$<10^{-4}$	$<10^{-4}$
4	745	1365661	88	266	282	$<10^{-4}$	$<10^{-4}$
5	882	1601648	182	315	330	$<10^{-4}$	$<10^{-4}$
6	1035	1735760	173	370	358	$<10^{-4}$	$<10^{-4}$
7	901	1609177	247	322	332	$<10^{-4}$	$<10^{-4}$
8	668	1135640	121	239	234	$<10^{-4}$	$<10^{-4}$
9	770	1382150	168	275	285	$<10^{-4}$	$<10^{-4}$
10	727	1322286	228	260	273	0.043101	0.004947
11	1278	2005315	604	457	414	$<10^{-4}$	$<10^{-4}$
12	1033	1776908	455	369	367	$<10^{-4}$	$<10^{-4}$
13	324	634435	140	116	131	0.027647	0.426854
14	614	1079560	194	219	223	0.092412	0.052193
15	589	1189858	222	210	245	0.400331	0.134979
16	858	1451775	322	307	300	0.381257	0.183929
17	1184	1971211	379	423	407	0.02731	0.160145
18	268	534152	103	96	110	0.47152	0.53236
19	1467	2277812	302	524	470	$<10^{-4}$	$<10^{-4}$
20	540	811690	165	193	167	0.040901	0.90657
21	233	342226	144	83	71	$<10^{-4}$	$<10^{-4}$
22	439	712404	46	157	147	$<10^{-4}$	$<10^{-4}$
X	840	1296174	1445	300	267	$<10^{-4}$	$<10^{-4}$
Y	45	67500	24	16	14	0.058372	0.01451

[a]results of binomial test

Table S15: Chromosomal distribution of variants in dataset DS16

chromosome	no. of genes	CDS length	no. of observed variants	no. of expected variants (no. of genes)	no. of expected variants (CDS length)	p-value[a] (no. of genes)	p-value[a] (CDS length)
1	2037	3483903	92	108	107	0.115079	0.138772
2	1238	2517642	92	66	77	0.001774	0.086338
3	1071	1965098	83	57	60	0.000809	0.00423
4	745	1365661	44	40	42	0.518289	0.693893
5	882	1601648	79	47	49	$<10^{-4}$	$<10^{-4}$
6	1035	1735760	84	55	53	0.00017	$<10^{-4}$
7	901	1609177	61	48	49	0.064099	0.093453
8	668	1135640	51	36	35	0.013807	0.009406
9	770	1382150	43	41	42	0.749604	0.875602
10	727	1322286	51	39	41	0.059765	0.108456
11	1278	2005315	47	68	62	0.006932	0.056584
12	1033	1776908	41	55	55	0.052282	0.060256
13	324	634435	15	17	19	0.714273	0.359795
14	614	1079560	42	33	33	0.111232	0.132256
15	589	1189858	38	31	37	0.201067	0.800186
16	858	1451775	33	46	45	0.049701	0.078171
17	1184	1971211	26	63	61	$<10^{-4}$	$<10^{-4}$
18	268	534152	21	14	16	0.077577	0.260035
19	1467	2277812	42	78	70	$<10^{-4}$	0.000314
20	540	811690	17	29	25	0.023161	0.126941
21	233	342226	13	12	11	0.77006	0.434565
22	439	712404	18	23	22	0.342093	0.515226
X	840	1296174	20	45	40	$<10^{-4}$	0.00065
Y	45	67500	0	2	2	0.277783	0.281607

[a]results of binomial test

Table S16: Chromosomal distribution of variants in dataset DS17

chromosome	no. of genes	CDS length	no. of observed variants	no. of expected variants (no. of genes)	no. of expected variants (CDS length)	p-value[a] (no. of genes)	p-value[a] (CDS length)
1	2037	3483903	26	77	76	$<10^{-4}$	$<10^{-4}$
2	1238	2517642	54	47	55	0.291052	0.944223
3	1071	1965098	62	41	43	0.001633	0.004585
4	745	1365661	7	28	30	$<10^{-4}$	$<10^{-4}$
5	882	1601648	15	33	35	0.000655	0.000179
6	1035	1735760	13	39	38	$<10^{-4}$	$<10^{-4}$
7	901	1609177	54	34	35	0.001079	0.002401
8	668	1135640	3	25	25	$<10^{-4}$	$<10^{-4}$
9	770	1382150	14	29	30	0.00311	0.001487
10	727	1322286	4	28	29	$<10^{-4}$	$<10^{-4}$
11	1278	2005315	39	49	44	0.159694	0.484416
12	1033	1776908	63	39	39	0.000267	0.000264
13	324	634435	7	12	14	0.187297	0.058034
14	614	1079560	11	23	24	0.007787	0.006197
15	589	1189858	28	22	26	0.193035	0.689412
16	858	1451775	64	33	32	$<10^{-4}$	$<10^{-4}$
17	1184	1971211	90	45	43	$<10^{-4}$	$<10^{-4}$
18	268	534152	8	10	12	0.633766	0.373646
19	1467	2277812	11	56	50	$<10^{-4}$	$<10^{-4}$
20	540	811690	11	20	18	0.040457	0.117439
21	233	342226	18	9	7	0.006227	0.000735
22	439	712404	1	17	16	$<10^{-4}$	$<10^{-4}$
X	840	1296174	148	32	28	$<10^{-4}$	$<10^{-4}$
Y	45	67500	0	2	1	0.27761	0.41331

[a]results of binomial test

Table S17: Chromosomal distribution of variants in dataset DS18

chromosome	no. of genes	CDS length	no. of observed variants	no. of expected variants (no. of genes)	no. of expected variants (CDS length)	p-value[a] (no. of genes)	p-value[a] (CDS length)
1	2037	3483903	1523	1653	1632	0.000699	0.004236
2	1238	2517642	920	1004	1179	0.005599	$<10^{-4}$
3	1071	1965098	1036	869	921	$<10^{-4}$	0.000116
4	745	1365661	566	604	640	0.115063	0.002641
5	882	1601648	654	716	750	0.018645	0.000265
6	1035	1735760	546	840	813	$<10^{-4}$	$<10^{-4}$
7	901	1609177	1307	731	754	$<10^{-4}$	$<10^{-4}$
8	668	1135640	420	542	532	$<10^{-4}$	$<10^{-4}$
9	770	1382150	528	625	647	$<10^{-4}$	$<10^{-4}$
10	727	1322286	433	590	619	$<10^{-4}$	$<10^{-4}$
11	1278	2005315	1010	1037	939	0.394838	0.018569
12	1033	1776908	861	838	832	0.414442	0.310314
13	324	634435	255	263	297	0.663235	0.012794
14	614	1079560	427	498	506	0.001043	0.000299
15	589	1189858	605	478	557	$<10^{-4}$	0.042692
16	858	1451775	670	696	680	0.323032	0.7097
17	1184	1971211	795	961	923	$<10^{-4}$	$<10^{-4}$
18	268	534152	231	217	250	0.356449	0.23832
19	1467	2277812	1016	1190	1067	$<10^{-4}$	0.109553
20	540	811690	278	438	380	$<10^{-4}$	$<10^{-4}$
21	233	342226	110	189	160	$<10^{-4}$	$<10^{-4}$
22	439	712404	267	356	334	$<10^{-4}$	$<10^{-4}$
X	840	1296174	1588	682	607	$<10^{-4}$	$<10^{-4}$
Y	45	67500	8	37	32	$<10^{-4}$	$<10^{-4}$

[a]results of binomial test

Table S18: Chromosomal distribution of variants in dataset DS19

chromosome	no. of genes	CDS length	no. of observed variants	no. of expected variants (no. of genes)	no. of expected variants (CDS length)	p-value[a] (no. of genes)	p-value[a] (CDS length)
1	2037	3483903	890	1057	1044	$<10^{-4}$	$<10^{-4}$
2	1238	2517642	498	642	754	$<10^{-4}$	$<10^{-4}$
3	1071	1965098	533	556	589	0.337193	0.017432
4	745	1365661	268	387	409	$<10^{-4}$	$<10^{-4}$
5	882	1601648	364	458	480	$<10^{-4}$	$<10^{-4}$
6	1035	1735760	380	537	520	$<10^{-4}$	$<10^{-4}$
7	901	1609177	518	467	482	0.017911	0.097593
8	668	1135640	243	347	340	$<10^{-4}$	$<10^{-4}$
9	770	1382150	298	400	414	$<10^{-4}$	$<10^{-4}$
10	727	1322286	359	377	396	0.358483	0.057909
11	1278	2005315	682	663	601	0.445497	0.000766
12	1033	1776908	611	536	532	0.001104	0.000606
13	324	634435	125	168	190	0.000533	$<10^{-4}$
14	614	1079560	288	319	323	0.082546	0.044844
15	589	1189858	333	306	356	0.116748	0.214921
16	858	1451775	377	445	435	0.000822	0.004137
17	1184	1971211	2254	614	591	$<10^{-4}$	$<10^{-4}$
18	268	534152	109	139	160	0.009148	$<10^{-4}$
19	1467	2277812	452	761	682	$<10^{-4}$	$<10^{-4}$
20	540	811690	193	280	243	$<10^{-4}$	$<10^{-4}$
21	233	342226	69	121	103	$<10^{-4}$	$<10^{-4}$
22	439	712404	95	228	213	$<10^{-4}$	$<10^{-4}$
X	840	1296174	326	436	388	$<10^{-4}$	0.001109
Y	45	67500	1	23	20	$<10^{-4}$	$<10^{-4}$

[a]results of binomial test

Table S19: Chromosomal distribution of variants in dataset DS20

chromosome	no. of genes	CDS length	no. of observed variants	no. of expected variants (no. of genes)	no. of expected variants (CDS length)	p-value[a] (no. of genes)	p-value[a] (CDS length)
1	2037	3483903	926	911	900	0.599798	0.351283
2	1238	2517642	516	554	650	0.099744	$<10^{-4}$
3	1071	1965098	490	479	507	0.605278	0.437009
4	745	1365661	280	333	353	0.002554	$<10^{-4}$
5	882	1601648	292	395	414	$<10^{-4}$	$<10^{-4}$
6	1035	1735760	450	463	448	0.550686	0.922761
7	901	1609177	253	403	416	$<10^{-4}$	$<10^{-4}$
8	668	1135640	281	299	293	0.316918	0.494541
9	770	1382150	289	344	357	0.002071	0.000172
10	727	1322286	264	325	341	0.00041	$<10^{-4}$
11	1278	2005315	677	572	518	$<10^{-4}$	$<10^{-4}$
12	1033	1776908	425	462	459	0.077036	0.108194
13	324	634435	143	145	164	0.933215	0.105772
14	614	1079560	254	275	279	0.220038	0.135891
15	589	1189858	238	263	307	0.117748	$<10^{-4}$
16	858	1451775	409	384	375	0.191867	0.07274
17	1184	1971211	584	530	509	0.016469	0.000788
18	268	534152	161	120	138	0.000325	0.053276
19	1467	2277812	542	656	588	$<10^{-4}$	0.049602
20	540	811690	197	242	210	0.0033	0.401441
21	233	342226	154	104	88	$<10^{-4}$	$<10^{-4}$
22	439	712404	208	196	184	0.386577	0.07977
X	840	1296174	815	376	335	$<10^{-4}$	$<10^{-4}$
Y	45	67500	2	20	17	$<10^{-4}$	$<10^{-4}$

[a]results of binomial test

Table S20: Chromosomal distribution of variants in dataset DS21

chromosome	no. of genes	CDS length	no. of observed variants	no. of expected variants (no. of genes)	no. of expected variants (CDS length)	p-value[a] (no. of genes)	p-value[a] (CDS length)
1	2037	3483903	4129	4158	4106	0.640751	0.704904
2	1238	2517642	2719	2527	2967	$<10^{-4}$	$<10^{-4}$
3	1071	1965098	2272	2186	2316	0.060073	0.351855
4	745	1365661	1241	1521	1610	$<10^{-4}$	$<10^{-4}$
5	882	1601648	1336	1800	1888	$<10^{-4}$	$<10^{-4}$
6	1035	1735760	1999	2113	2046	0.010838	0.296551
7	901	1609177	1558	1839	1897	$<10^{-4}$	$<10^{-4}$
8	668	1135640	998	1364	1338	$<10^{-4}$	$<10^{-4}$
9	770	1382150	1298	1572	1629	$<10^{-4}$	$<10^{-4}$
10	727	1322286	1226	1484	1558	$<10^{-4}$	$<10^{-4}$
11	1278	2005315	3107	2609	2363	$<10^{-4}$	$<10^{-4}$
12	1033	1776908	1821	2109	2094	$<10^{-4}$	$<10^{-4}$
13	324	634435	797	661	748	$<10^{-4}$	0.070451
14	614	1079560	1338	1253	1272	0.015926	0.062043
15	589	1189858	1238	1202	1402	0.298585	$<10^{-4}$
16	858	1451775	1762	1751	1711	0.78814	0.207681
17	1184	1971211	2286	2417	2323	0.005802	0.435357
18	268	534152	730	547	630	$<10^{-4}$	$<10^{-4}$
19	1467	2277812	2498	2995	2685	$<10^{-4}$	0.000173
20	540	811690	967	1102	957	$<10^{-4}$	0.731147
21	233	342226	560	476	403	$<10^{-4}$	$<10^{-4}$
22	439	712404	776	896	840	$<10^{-4}$	0.02676
X	840	1296174	3704	1715	1528	$<10^{-4}$	$<10^{-4}$
Y	45	67500	29	92	80	$<10^{-4}$	$<10^{-4}$

[a]results of binomial test

Table S21: Chromosomal distribution of variants in dataset DS22

chromosome	no. of genes	CDS length	no. of observed variants	no. of expected variants (no. of genes)	no. of expected variants (CDS length)	p-value[a] (no. of genes)	p-value[a] (CDS length)
1	2037	3483903	2272	2173	2146	0.025674	0.004253
2	1238	2517642	1332	1321	1551	0.743783	$<10^{-4}$
3	1071	1965098	1153	1143	1210	0.749404	0.091476
4	745	1365661	798	795	841	0.899286	0.134722
5	882	1601648	989	941	986	0.109369	0.922068
6	1035	1735760	1656	1104	1069	$<10^{-4}$	$<10^{-4}$
7	901	1609177	846	961	991	0.000119	$<10^{-4}$
8	668	1135640	665	713	699	0.070241	0.19102
9	770	1382150	770	821	851	0.069462	0.004114
10	727	1322286	861	776	814	0.002111	0.096521
11	1278	2005315	1493	1363	1235	0.000337	$<10^{-4}$
12	1033	1776908	922	1102	1094	$<10^{-4}$	$<10^{-4}$
13	324	634435	303	346	391	0.01968	$<10^{-4}$
14	614	1079560	677	655	665	0.382461	0.622311
15	589	1189858	607	628	733	0.406319	$<10^{-4}$
16	858	1451775	815	915	894	0.000602	0.00658
17	1184	1971211	1115	1263	1214	$<10^{-4}$	0.003261
18	268	534152	384	286	329	$<10^{-4}$	0.002684
19	1467	2277812	1610	1565	1403	0.237109	$<10^{-4}$
20	540	811690	591	576	500	0.526241	$<10^{-4}$
21	233	342226	275	249	211	0.096966	$<10^{-4}$
22	439	712404	564	468	439	$<10^{-4}$	$<10^{-4}$
X	840	1296174	406	896	798	$<10^{-4}$	$<10^{-4}$
Y	45	67500	3	48	42	$<10^{-4}$	$<10^{-4}$

[a]results of binomial test

Table S22: Chromosomal distribution of variants in dataset DS23

chromosome	no. of genes	CDS length	no. of observed variants	no. of expected variants (no. of genes)	no. of expected variants (CDS length)	p-value[a] (no. of genes)	p-value[a] (CDS length)
1	2037	3483903	2061	2285	2256	$<10^{-4}$	$<10^{-4}$
2	1238	2517642	1603	1388	1630	$<10^{-4}$	0.495313
3	1071	1965098	1260	1201	1272	0.08262	0.739836
4	745	1365661	509	836	884	$<10^{-4}$	$<10^{-4}$
5	882	1601648	486	989	1037	$<10^{-4}$	$<10^{-4}$
6	1035	1735760	753	1161	1124	$<10^{-4}$	$<10^{-4}$
7	901	1609177	821	1011	1042	$<10^{-4}$	$<10^{-4}$
8	668	1135640	412	749	735	$<10^{-4}$	$<10^{-4}$
9	770	1382150	624	864	895	$<10^{-4}$	$<10^{-4}$
10	727	1322286	491	815	856	$<10^{-4}$	$<10^{-4}$
11	1278	2005315	1959	1433	1299	$<10^{-4}$	$<10^{-4}$
12	1033	1776908	1015	1159	1151	$<10^{-4}$	$<10^{-4}$
13	324	634435	533	363	411	$<10^{-4}$	$<10^{-4}$
14	614	1079560	741	689	699	0.044082	0.106484
15	589	1189858	698	661	770	0.138545	0.007424
16	858	1451775	1059	962	940	0.001643	$<10^{-4}$
17	1184	1971211	1280	1328	1276	0.178821	0.908187
18	268	534152	388	301	346	$<10^{-4}$	0.024453
19	1467	2277812	1069	1645	1475	$<10^{-4}$	$<10^{-4}$
20	540	811690	441	606	526	$<10^{-4}$	0.000146
21	233	342226	311	261	222	0.002529	$<10^{-4}$
22	439	712404	256	492	461	$<10^{-4}$	$<10^{-4}$
X	840	1296174	3395	942	839	$<10^{-4}$	$<10^{-4}$
Y	45	67500	26	50	44	$<10^{-4}$	0.005013

[a]results of binomial test

Table S23: Chromosomal distribution of variants in dataset DS24

chromosome	no. of genes	CDS length	no. of observed variants	no. of expected variants (no. of genes)	no. of expected variants (CDS length)	p-value[a] (no. of genes)	p-value[a] (CDS length)
1	2037	3483903	7056	7702	7605	$<10^{-4}$	$<10^{-4}$
2	1238	2517642	5323	4681	5496	$<10^{-4}$	0.015334
3	1071	1965098	4293	4050	4290	$<10^{-4}$	0.956108
4	745	1365661	2362	2817	2981	$<10^{-4}$	$<10^{-4}$
5	882	1601648	2710	3335	3496	$<10^{-4}$	$<10^{-4}$
6	1035	1735760	4911	3913	3789	$<10^{-4}$	$<10^{-4}$
7	901	1609177	3225	3407	3513	0.001371	$<10^{-4}$
8	668	1135640	2211	2526	2479	$<10^{-4}$	$<10^{-4}$
9	770	1382150	2583	2911	3017	$<10^{-4}$	$<10^{-4}$
10	727	1322286	2539	2749	2887	$<10^{-4}$	$<10^{-4}$
11	1278	2005315	5151	4832	4378	$<10^{-4}$	$<10^{-4}$
12	1033	1776908	3396	3906	3879	$<10^{-4}$	$<10^{-4}$
13	324	634435	1506	1225	1385	$<10^{-4}$	0.001188
14	614	1079560	2119	2322	2357	$<10^{-4}$	$<10^{-4}$
15	589	1189858	2304	2227	2597	0.099801	$<10^{-4}$
16	858	1451775	3467	3244	3169	$<10^{-4}$	$<10^{-4}$
17	1184	1971211	5238	4477	4303	$<10^{-4}$	$<10^{-4}$
18	268	534152	1088	1013	1166	0.019239	0.021306
19	1467	2277812	4452	5547	4973	$<10^{-4}$	$<10^{-4}$
20	540	811690	1672	2042	1772	$<10^{-4}$	0.016196
21	233	342226	856	881	747	0.406342	$<10^{-4}$
22	439	712404	1430	1660	1555	$<10^{-4}$	0.001241
X	840	1296174	4883	3176	2830	$<10^{-4}$	$<10^{-4}$
Y	45	67500	37	170	147	$<10^{-4}$	$<10^{-4}$

[a]results of binomial test

Table S24: Distribution of variants to chromosomes in the datasets

Dataset	Pearson χ^2 test statistic	p-value
DS1	8657.11	$<10^{-4}$
DS2	971.29	$<10^{-4}$
DS3	13522.20	$<10^{-4}$
DS4	689.31	$<10^{-4}$
DS5	7949.79	$<10^{-4}$
DS6	1180.34	$<10^{-4}$
DS7	14122.93	$<10^{-4}$
DS8	928.41	$<10^{-4}$
DS9	8287.18	$<10^{-4}$
DS10	522.68	$<10^{-4}$
DS11	7405.09	$<10^{-4}$
DS12	210.97	$<10^{-4}$
DS13	917.23	$<10^{-4}$
DS14	370.89	$<10^{-4}$
DS15	5210.03	$<10^{-4}$
DS16	158.15	$<10^{-4}$
DS17	740.94	$<10^{-4}$
DS18	2141.00	$<10^{-4}$
DS19	4946.55	$<10^{-4}$
DS20	746.13	$<10^{-4}$
DS21	3147.36	$<10^{-4}$
DS22	757.39	$<10^{-4}$
DS23	8068.18	$<10^{-4}$
DS24	2363.46	$<10^{-4}$
